# Unraveling endometriosis-associated ovarian carcinomas using integrative proteomics

**DOI:** 10.12688/f1000research.13863.2

**Published:** 2018-06-20

**Authors:** Felix Leung, Marcus Q. Bernardini, Kun Liang, Ihor Batruch, Marjan Rouzbahman, Eleftherios P. Diamandis, Vathany Kulasingam

**Affiliations:** 1 Department of Laboratory Medicine and Pathobiology, University of Toronto, Toronto, Ontario, M5S 1A8, Canada; 2 Department of Obstetrics and Gynecology, University of Toronto, Toronto, Ontario, M5G 1E2, Canada; 3 Department of Statistics and Actuarial Science, University of Waterloo, Waterloo, Ontario, N2L 3G1 , Canada; 4 Department of Pathology and Laboratory Medicine, Mount Sinai Hospital, Toronto, Ontario, M5T 3L9, Canada; 5 Department of Pathology, Laboratory Medicine Program, University Health Network, Toronto, Ontario, M5G 2C4, Canada; 6 Department of Clinical Biochemistry, University Health Network, Toronto, Ontario, M5G 2C4, Canada

**Keywords:** ovarian cancer, endometriosis, clear cell carcinoma, endometrioid carcinoma, proteomics, bioinformatics

## Abstract

**Background:** To elucidate potential markers of endometriosis and endometriosis-associated endometrioid and clear cell ovarian carcinomas using mass spectrometry-based proteomics.

**Methods:** A total of 21 fresh, frozen tissues from patients diagnosed with clear cell carcinoma, endometrioid carcinoma, endometriosis and benign endometrium were subjected to an in-depth liquid chromatography-tandem mass spectrometry analysis on the Q-Exactive Plus. Protein identification and quantification were performed using MaxQuant, while downstream analyses were performed using Perseus and various bioinformatics databases.

**Results: **Approximately 9000 proteins were identified in total, representing the first in-depth proteomic investigation of endometriosis and its associated cancers. This proteomic data was shown to be biologically sound, with minimal variation within patient cohorts and recapitulation of known markers. While moderate concordance with genomic data was observed, it was shown that such data are limited in their abilities to represent tumours on the protein level and to distinguish tumours from their benign precursors.

**Conclusions:** The proteomic data suggests that distinct markers may differentiate endometrioid and clear cell carcinoma from endometriosis. These markers may be indicators of pathobiology but will need to be further investigated. Ultimately, this dataset may serve as a basis to unravel the underlying biology of the endometrioid and clear cell cancers with respect to their endometriotic origins.

## Introduction

Ovarian cancer (OvCa) is not a single disease but is made up of several distinct subtypes, including serous, endometrioid, clear cell and mucinous. It is now accepted that the majority of serous OvCa arise from carcinomas of the fallopian tube secretory epithelium due to histopathological evidence ( Kurman & Shih, 2016). Unfortunately, the origins of the endometrioid, clear cell and mucinous subtypes are not well-delineated and these subtypes remain poorly understood. Genomic and morphologic studies have identified links between endometriosis lesions that progress to endometrioid and clear cell OvCa ( Prowse *et al.*, 2006; Wang *et al.*, 2015). Of note, *ARID1A* and *PTEN/PIK3CA* mutations have been identified as hallmark features of endometriosis-associated OvCa ( Jones *et al.*, 2010; Kuo *et al.*, 2009; Wiegand *et al.*, 2014); however, none of these associations have been characterized at the proteomic level and the mechanisms driving tumourigenesis in these precursors have yet to be identified. As such, proteomic profiling may aid in substantiating these purported precursors of non-serous OvCa, as well as in revealing the underlying biology behind why these seemingly distinct diseases converge on the ovaries upon metastasis and clinical presentation.

Proteomic profiling of OvCa has mainly revolved around mass spectrometry (MS)-based analyses. The study of protein expression in OvCa has been increasingly important due to the central role of proteins in all biological processes. Moreover, the proteome integrates the cellular genetic information and environmental influences. As such, MS has been increasingly implemented as it allows for simultaneous examination of thousands of proteins in biospecimens. With respect to endometriosis-associated OvCa, there exist limited studies investigating the diseases on a proteomic level. One recent study utilized proteomic approaches to characterize *ARID1A* and *PIK3CA* mutations in endometriosis-associated clear cell and endometrioid OvCa ( Wiegand *et al.*, 2014), but currently, there are no studies aimed at comprehensive proteomic profiling of these cancers and their suspected endometriotic lesions.

To this end, an in-depth proteomic analysis was performed on gynaecological tissue specimens using a label-free, liquid chromatography-tandem mass spectrometry (LC/MS-MS) method. Specifically, tissue proteomes of the following specimens were delineated: clear cell ovarian carcinoma (CC); endometrioid ovarian carcinoma (EC); endometriosis (EMT); and benign endometrium (END). This exercise has identified approximately 9000 unique proteins and represents one of the most comprehensive proteomes of endometriosis and its associated cancers to date. These discovery data may serve as the basis to identifying markers of disease as well as understanding the pathobiology for these endometriosis-associated ovarian cancers.

## Methods

### Gynaecological Tissue Cohort

Gynaecological tissue samples from a total of 21 patients were retrospectively identified and selected for proteomic analysis. All samples were collected at University Health Network, Toronto, Canada (UHN REB Number 13-6360-CE) and immediately stored in liquid nitrogen until retrieval. Approximately 1 cm ^3^ of each fresh, frozen sample was retrieved for proteomic analysis. All samples were histopathologically confirmed for their diagnoses and tissue purity (at least 70%) by a gynaecologic pathologist using matched formalin-fixed, paraffin-embedded tissue slides. With respect to the histopathological diagnoses: six cases of CC, seven cases of EC, three cases of EMT and five cases of END were identified. A detailed description of clinical and histological characteristics of the patients can be found in Table 1.

**Table 1.** Clinical and histological characteristics of the gynecological patients.

Cohort	Age at Diagnosis (years)	Histology	Tumour Stage	Pre-surgical CA125 (IU/mL)
**Clear Cell Carcinoma**				
CC 1	57	Clear cell	IC	41
CC 7	56	Clear cell	IIC	1258
CC 8	59	Clear cell	IA	175
CC 9	74	Clear cell	IA	60
CC 10	58	Clear cell	IIB	117
CC 11	67	Clear cell	IIB	3135
**Endometrioid Carcinoma**				
EC 3	71	Endometrioid	IA	9305
EC 18	61	Endometrioid	IIB	110
EC 19	52	Endometrioid	IIIB	92
EC 20	40	Endometrioid	IA	361
EC 21	46	Endometrioid	IC	11
EC 22	60	Endometrioid	IA	58
EC 23	60	Endometrioid	IIA	204
**Endometriosis**				
EMT 4	49	Tubal endometriosis	-	75
EMT 24	49	Tubal endometriosis	-	49
EMT 25	46	Ovarian endometriosis	-	64
**Benign Endometrium**				
END 5	59	Secretory phase endometrium	-	-
END 26	72	Atrophic endometrium	-	7021
END 28	39	Proliferative endometrium	-	19
END 29	38	Proliferative endometrium	-	107
END 30	49	Atrophic endometrium	-	58

### Tissue Sample Preparation

Fresh, frozen samples were first grinded with 0.05% RapiGest (Waters, MA, USA) in 50 mM ammonium bicarbonate. The tissue mixtures were then homogenized and sonicated in order to disrupt cell membranes. This was followed by two rounds of centrifugation at 16200 *x g* for 30 minutes at 4°C and collection of the supernatant. Total protein concentration of the supernatant was determined using the Bradford protein assay. After adjusting for 1 mg of total protein content, each sample was subjected to reduction with 15 mM dithiothreitol (Sigma-Aldrich, ON, Canada) in 50 mM ammonium bicarbonate at 60°C for 30 minutes, followed by alkylation with 15 mM iodoacetamide (Sigma-Aldrich, ON, Canada) in 50 mM ammonium bicarbonate for 45 minutes in the dark at room temperature. Protein digestion was carried out with trypsin (Sigma-Aldrich, ON, Canada) in 50 mM ammonium bicarbonate (1:50 trypsin to total protein ratio) overnight at 37°C. RapiGest and trypsin digestion were stopped with the addition of 1% trifluoroacetic acid followed by centrifugation at 16200 *x g* for 30 minutes at 4°C. Digested samples were immediately frozen at -80°C until all samples were ready for fractionation via high-performance liquid chromatography (HPLC) – using strong cation exchange (SCX) columns – to reduce peptide complexity.

### SCX HPLC

Trypsinized samples were diluted two-fold in mobile phase A (0.26 M formic acid, 5% acetonitrile, pH 2–3) and loaded directly onto a 500 μL loop connected to a PolySULFOETHYL A Column (2.1 mm × 200 mm; 5 μ; 200 Å; The Nest Group, Inc., MA, USA), containing a silica-based hydrophilic, anionic polymer (poly-2-sulfoethyl aspartamide). The Agilent 1100 HPLC system (Agilent Technologies, Germany) was used for SCX peptide fractionation. A 60-minute gradient was employed with a gradual increase of mobile phase B (0.26 M formic acid, 5% acetonitrile, 1 M ammonium formate, pH 4–5) starting at 30 minutes (30–40 minutes 20% mobile phase B; 40–55 minutes 100% mobile phase B) for the elution of peptides at a flow rate of 200 μL/minute. The eluent was monitored at a wavelength of 280 nm and fractions were collected every three minutes from 28 to 55 minutes resulting in a total of 9 fractions per sample. SCX column and system performance were assessed by running a quality control peptide mixture consisting of 1 μg/μL alpha bag cell peptide, 1 μg/μL fibrinogen fragment, 5 μg/μL human adrenocorticotropic hormone, and 5 μg/μL angiotensin-converting enzyme inhibitor (American Protein Company, CA) after every biological sample.

### LC-MS/MS

The SCX fractions were purified through C-18 OMIX Pipette Tips (Agilent Technologies, Germany) to remove impurities and salts as well as to resuspend the tryptic peptides in a buffer compatible with the mass spectrometer. The fractions were eluted in 5 μL of 65% MS buffer B (90% acetonitrile, 0.1% formic acid, 10% water, 0.02% trifluoroacetic acid) and 35% MS buffer A (5% acetonitrile, 0.1% formic acid, 95% water, 0.02% trifluoroacetic acid). Using an auto-sampler, 18 µL of each sample were injected into an in-house packed 3.3 cm trap pre-column (5 μm C18 particle, column inner diameter 150 μm) and peptides were eluted from the 15 cm analytical column (3 μm C18 particle, inner diameter 75 μm, tip diameter 8 μm). The LC EASY-nLC system (Thermo Fisher, Denmark) was coupled online to the Q-Exactive Plus (Thermo Fischer, CA, USA) mass spectrometer with a nanoelectrospray ionization source. A 60 min LC gradient was applied with an increasing percentage of MS buffer B for peptide elution at a flow rate of 300 nL/min. Full MS1 scan was acquired from a scan range of 400–1500 m/z in the Orbitrap at a resolution of 70000, followed by the MS2 scans for the top 12 precursor ions at a resolution of 17500 in a data-dependent acquisition mode and isolation window of 1.6 m/z. The dynamic exclusion was enabled for 45 seconds and unassigned charge, as well as charge states +1 and +4 to ≥8 were omitted from MS2 fragmentation. Each biological sample was separated into nine fractions with each fraction being subjected to a 60 minute LC gradient. To minimize for instrumentation bias, samples were run in batches with each batch containing approximately one patient from each disease cohort. Quality controls were run between each biological sample (before and after all nine fractions from a sample) as well as between batches to ensure consistent machine performance for all samples.

### Protein identification and label-free quantification

RAW files were uploaded into MaxQuant ver. 1.5.2.8 ( Cox & Mann, 2008) and searched with Andromeda (built into MaxQuant) against the human UniProtKB/Swiss-Prot database (January 2015 release; 550299 sequence entries). Search parameters included a fixed carbamidomethylation of cysteines and variable modifications of methionine oxidation and N-terminal acetylation. Data was initially searched against a “human first search” database with a parent tolerance of 20 ppm and a fragment tolerance of 0.5 Da in order to calculate and adjust the correct parent tolerance to 5 ppm for the search against the UniProtKB/Swiss-Prot database. During the search, the UniProtKB/Swiss-Prot database was randomized and false detection rate was set to 1% at the peptide and protein levels. Data was analyzed using “Label-free quantification” checked, and the “Match between runs” interval was set to 2 minutes. Proteins identified with a minimum of two unique peptides were selected for further statistical analyses. “LFQ Intensity” columns corresponding to the extracted ion current value of each protein were used for further statistical analyses to determine overexpressed proteins.

The produced MaxQuant output matrix was loaded onto the Perseus software (ver. 1.5.2.6) to perform statistical and bioinformatics analyses ( Tyanova *et al.*, 2016). Specifically, LFQ data were logarithmically-transformed and imputed by creating a Gaussian distribution of random numbers with a standard deviation of 30% relative to the standard deviation of the measured values and one standard deviation down-shift of the mean to simulate the distribution of low signal values. Hierarchical clustering of proteins was performed on logarithmized intensities after z-score normalization of the data, using Euclidean distances. PCA was performed on logarithmized values using singular value decomposition in order to find the principal components. Gene Ontology, the Protein Analysis Through Evolutionary Relationships (PANTHER) Classification System ( Mi *et al.*, 2013), and ConsensusPathDB-human ( Kamburov *et al.*, 2009) were utilized to retrieve additional annotations.

### Statistical analysis

Quantitative protein data were log-transformed before statistical analyses. Proteins with missing values (‘0’ normalized values) were imputed using the Perseus software. The elevated protein levels were detected using LIMMA, an empirical Bayes method proposed by Smyth ( Smyth, 2004). The posterior probability of being elevated for each protein was computed using the two-group model ( Efron & Tibshirani, 2002) with true null probability estimated by the right-boundary procedure ( Liang & Nettleton, 2012). Protein levels in control tissues were assumed to follow normal distributions and estimated their means and variances conservatively.

## Results

### Deep proteomic analysis of tissue specimens

**Figure 1.**
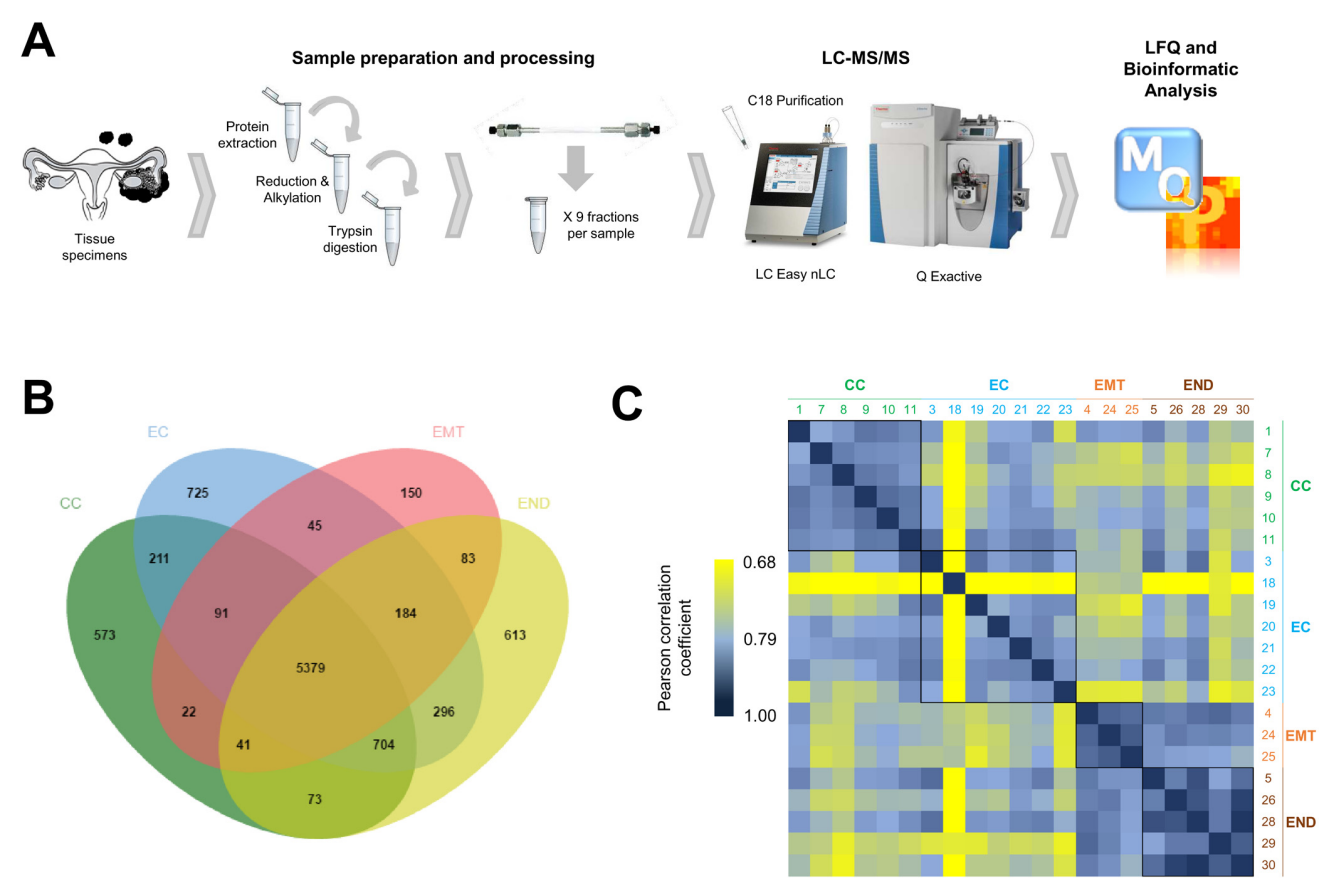
Proteomic workflow and dataset. ( **A**) Overview of the label-free LC-MS/MS workflow developed for proteomic analysis of tissue specimens. ( **B**) Venn diagram displaying overlap of proteins between the four patient cohorts. ( **C**) Interpatient correlation of the protein expression profiles.

To decipher the in-depth proteome of the tissues, we utilized an offline two-dimensional LC-MS/MS workflow amenable to label-free quantification (LFQ) with minimal amount of protein required upfront (1mg total protein). As seen in Figure 1A, lysis, reduction and alkylation, and trypsin digestion were performed in a single-tube manner prior to SCX-HPLC. The additional offline SCX-HPLC coupled with online C18 reverse phase chromatography fractionation and MS/MS analysis on the Q-Exactive Plus ensured comprehensive proteome coverage and high mass accuracy.

Overall, 8793 unique proteins were identified across the 21 patient samples with roughly 5000 unique proteins identified within each patient cohort, as seen in Supplementary Figure 1. In terms of overlap, 5379 proteins were identified in all four cohorts ( Figure 1B). Approximately 90% of all proteins were identified with at least 2 or more peptides ( Supplementary Figure 2). With respect to protein expression patterns, strong correlations between samples within the same patient cohort were observed. As seen in Figure 1C, protein expression was relatively consistent when comparing patients with the same diagnosis with Pearson correlation coefficients (PCC) above approximately 0.80 for CC, EC, EMT and END. A notable exception was patient EC 18 who displayed decreased correlation with the other EC patients with PCC ≤0.72. Interestingly, subsequent re-examination of Patient 18 revealed more mucinous-like histological features and it was concluded that the final diagnosis of EC (as opposed to mucinous carcinoma) was due to the lack of metastatic gastrointestinal involvement (data not shown). Patient 18 was removed from further analyses given its mucinous-like features, thus making it less likely to be an endometriosis-associated cancer. The biological soundness of the proteomic data was further demonstrated by the stable expression of housekeeping proteins, such as ribosomal biogenesis and assembly proteins, across all samples ( Supplementary Figure 3). Collectively, the minimal variation with respect to patient cohort and housekeeping protein expression (despite being processed in different batches) suggests that any differences observed in this dataset are due to true biological differences and not from technical artefacts.

### Correlation with clinical markers

**Figure 2.**
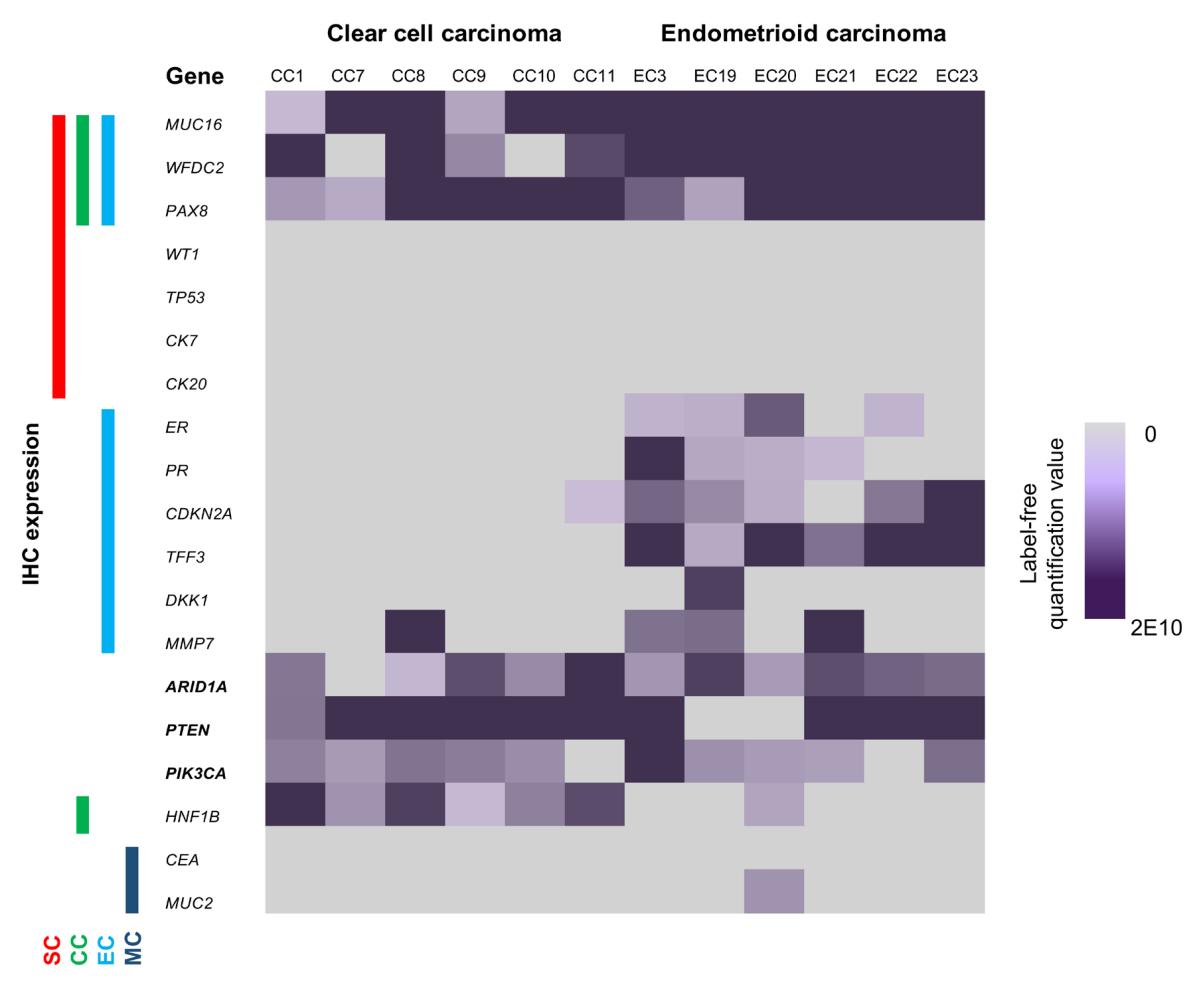
Correlation with immunohistochemical (IHC) markers. An expression matrix demonstrating the correlation of proteomic data with known IHC markers. Label-free quantification values (in arbitrary units represented by the purple gradient scale) were generated from MaxQuant and known IHC markers across the various subtypes were based on collating literature evidence. The expression of the markers across the serous (SC), clear cell (CC), endometrioid (EC) and mucinous (MC) subtypes are denoted by the red, green, blue and indigo lines, respectively.

As an initial assessment of the accuracy of proteomic profiling, we investigated the expression of various immunohistochemical (IHC) markers used in histopathological analysis of endometriosis-associated ovarian cancers ( Kalloger *et al.*, 2011; Köbel *et al.*, 2008; Köbel *et al.*, 2014). A spectrum of markers including general epithelial ovarian carcinoma markers and markers specific to various subtypes (serous, endometrioid, clear cell, and mucinous) were identified with the full panel of markers summarized in Figure 2. Overall, the proteomic data across the six CC and six EC samples correlated well with IHC expression based on literature evidence. Serous-specific markers such as Wilm’s Tumour 1 ( *WT1*), p53 ( *TP53*), cytokeratin 7 ( *CK7*) and cytokeratin 20 ( *CK20*) and mucinous-specific markers such as carcinoembryonic antigen ( *CEA*) and mucin 2 ( *MUC2*) were found to be expressed in almost none of the CC or EC samples. Meanwhile, EC-specific markers such as estrogen receptor ( *ER*), progesterone receptor ( *PR*), p16 ( *CDKN2A*), trefoil factor 3 ( *TFF3*), Dickkopf-related protein 1 ( *DKK1*), and matrix metalloproteinase 7 ( *MMP7*) and the CC-specific marker hepatocyte nuclear factor 1β ( *HNF1B*) displayed near exclusive expression in their respective subtypes. Expression of these markers in endometriotic and benign endometrial tissues was mostly low with the exception of *ER* and *PR* which were found to be constitutively expressed in the benign endometrial tissues (data available in “MaxQuant Analysis” dataset).

Interestingly, mucin 16 ( *MUC16*) and WAF four-disulfide core domain protein 2 ( *WFDC2*) displayed high overall expression in the EC cohort and variable expression in the CC cohort. Mucin 16 (also known as CA125) and WAF four-disulfide core domain protein 2 (also known as HE4) are the only clinically-approved serum markers of ovarian cancer and have been shown to perform better for serous OvCa compared to other subtypes ( Leung *et al.*, 2016; Shen *et al.*, 2017). Here, our data suggests that serum levels of such tumour markers ( Table 1) do not entirely capture the biology of the actual tumours and may actually be dependent on tumour release of the markers into circulation rather than tumour production.

Furthermore, protein expression of *ARID1A*, *PTEN*, and *PIK3CA* was investigated as mutations in these genes are often prevalent in endometriosis-associated cancers and have been suggested to be involved with genomic instability and tumourigenesis ( Jones *et al.*, 2010; Kuo *et al.*, 2009; McConechy *et al.*, 2014; Wiegand *et al.*, 2014; Yamamoto *et al.*, 2012). Despite genomic data that suggests concurrent loss of tumour suppressors *ARID1A* and *PTEN* with activating mutations of *PIK3CA* are involved in the pathogenesis of CC and EC, our data demonstrates that these genomic findings do not necessarily translate at the protein level. Indeed, variable expression of the three proteins is observed across the CC and EC cohorts. However, it is important to note that expression of these proteins does not imply proper functionality and thus, they may still contribute to tumourigenesis due to deleterious mutations.

### Mapping the CC- and EC-specific proteomes

**Figure 3.**
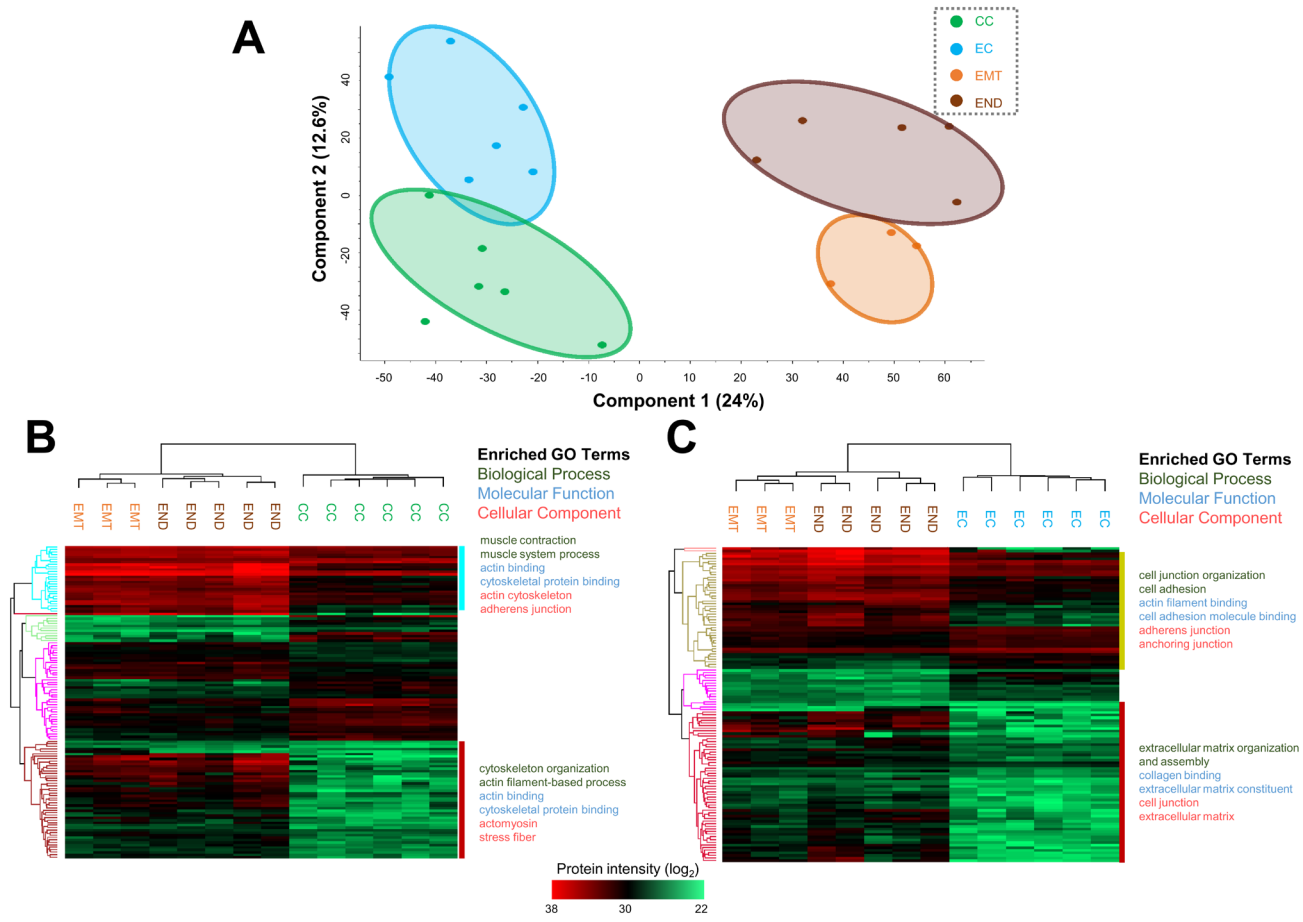
Principal component and clustering analyses. ( **A**) Principal component analysis of entire proteomic dataset without enrichment. Hierarchical clustering of differentially-expressed proteins between CC, EMT and END ( **B**) and EC, EMT and END ( **C**) with overrepresented GO annotations identified through enrichment analysis.

To evaluate how biological differences between tumours (CC or EC) and their precursors (EMT and END) were reflected at the proteomic level, unsupervised clustering was performed using the entire proteomic dataset without *a priori* enrichment. Using normalized log _2 _expression values, samples were analyzed using principal component analysis (PCA) to visualize the clustering of samples from the same cohorts as well as the separation between the cohorts. As expected, PCA revealed that in both comparisons of CC to EMT and END, and EC to EMT and END, samples within a cohort clustered together to produce clear separation between the cohorts with respect to overall protein expression ( Figures 3A). The strong segregation between the END, EMT and CC/EC cohorts suggested, once again, that differences between the cohorts are due to biological variation and that inspection of the proteomic landscape recapitulated these differences very well.

To elucidate the biological mechanisms contributing to the differences between tumours and their precursors, proteins demonstrating the highest variances between the cohorts had to be enriched for first. Using an ANOVA test with a Bonferroni-Hochberg FDR of 0.01, 127 proteins for the comparison of CC versus EMT/END and 119 proteins for the comparison of EC versus EMT/END were identified as having the strongest differential expression between the cohorts ( Supplementary Table 1). Subsequently, unsupervised hierarchical clustering was performed using these differentially-expressed proteins to verify the ability to distinguish between the cohorts, as well as to identify any notable clusters of differential expression patterns as seen in Figures 3B and 3C. As expected, the differential proteins were able to accurately separate all of the cohorts and produced distinct patterns of differential expression between tumours and their precursors. To identify overrepresented ontologies within these patterns of differential expression, enrichment analysis for Gene Ontology annotations was performed using the PANTHER Classification System. For CC, two major clusters were found to be characterized by predominantly muscle- and cytoskeletal-related processes ( Figure 3B), while for EC, two major clusters were found to be characterized by cell junction- and extracellular matrix-related processes ( Figure 3C). A condensed list of overrepresented annotations in both cancers are displayed in Supplementary Table 2.

### Integrating proteomic with genomic data

**Figure 4.**
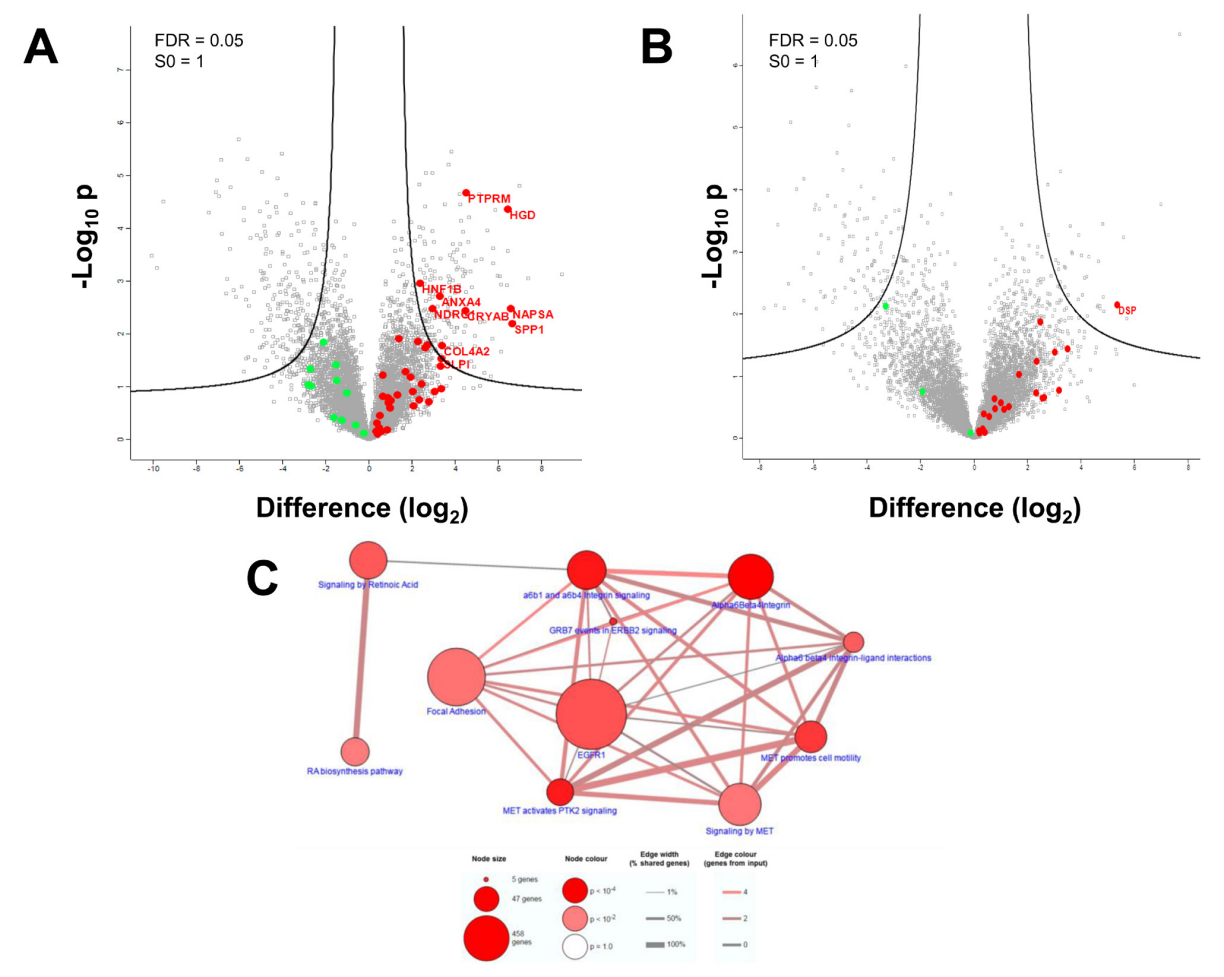
Integration with genomics and disease signature analyses. Volcano plot of CC versus EMT proteomes ( **A**) and EC versus EMT proteomes ( **B**) overlaid with concordant genomic features. The black lines denote statistical significance. ( **C**) depicts overrepresented pathways identified in the ‘disease signature’ derived for CC.

To assess concordance with existing genomic data, the proteomic dataset was compared against reported ‘subtype-specific gene signatures’ for CC and EC in previous RNA expression studies. For CC, a 113-gene signature that was reported to be able to differentiate between CC and high-grade serous OvCa was used ( Hughes *et al.*, 2016) while for EC, two studies comparing EC against high-grade serous OvCa were used after identifying 15 underexpressed genes and 40 overexpressed genes in EC ( Banz *et al.*, 2010; Uehara *et al.*, 2015). Of the 113 CC-specific genes, 11/34 of the underexpressed genes were downregulated at the proteomic level while 40/79 overexpressed genes were upregulated at the proteomic level ( Figure 4A). It is important to note that 10 of the 40 concordant genes were significantly upregulated in our data (FDR = 0.05, S0 = 1) with many of them being known markers of CC including napsin A (NAPSA), annexin 4 (ANXA4) and hepatocyte nuclear factor 1-beta (HNF1B). Furthermore, cystathionine gamma-lyase (CTH) and interleukin-6 receptor subunit beta (IL6RB) – markers that have been associated with CC ( Cochrane *et al.*, 2017; Hughes *et al.*, 2016; Yanaihara *et al.*, 2016) – were found to be elevated in CC compared to EMT samples. CTH was found to be significantly elevated in CC compared to EMT (p=0.01) while IL6RB was undetectable in all EMT samples but expressed in 4/6 of the CC samples. For EC, 3/15 of the underexpressed genes were downregulated at the proteomic level while 21/40 overexpressed genes were upregulated at the proteomic level ( Figure 4B). Unlike the observations in CC, only one of the concordant proteins (desmoplakin or DSP) was significantly upregulated. Interestingly, the concordant genes for both CC and EC were able to discriminate cancer from control patients at the proteomic level but could not distinguish EMT from END patients ( Supplementary Figure 4).

To further assess how well existing genomic data translates at the proteomic level, ARID1A and PIK3CA/PTEN-related proteins were inspected for their expression levels as these pathways are often perturbed in EC and CC. Overall, 21/37 ARID1A components and 49/254 PIK3CA/PTEN components were identified in our proteomic data (components identified using Reactome; http://www.reactome.org/). Unfortunately, these pathway-related proteins were poor discriminators and did not show obvious differential expression between the cancer and control cohorts ( Supplementary Figure 5), further highlighting that fact that genomic features do not always translate at the proteomic level.

### Identifying potential disease markers

In order to identify potential disease markers on a proteomic level, proteins with progressively increased expression from END to EMT to CC/EC were identified using LIMMA (see Materials & Methods for details). Using the following criteria: (A) progressively increased expression from END to EMT to EC/CC; (B) only increased expression from END to EMT; and (C) only increased expression from EMT to EC/CC, 25 proteins for EC and 252 proteins for CC were identified as ‘disease markers’ ( Supplementary Table 3– Supplementary Table 4). These disease marker signatures were able to discriminate between the different disease cohorts more accurately than the genomic signatures, especially with respect to differentiating between EMT and END patients ( Supplementary Figure 6). Further analysis of the CC signature revealed that there was an overrepresentation of pathways involving hepatocyte growth factor receptor (MET), α6β4 integrin, and retinoic acid ( Figure 4C). Pathway analysis was not performed for the EC signature due to a low number of proteins.

MaxQuant Analysis Guide An interpretive guide for the spreadsheet output from the MaxQuant analysis. Click here for additional data file. Copyright: © 2018 Leung F et al. 2018 Data associated with the article are available under the terms of the Creative Commons Zero "No rights reserved" data waiver (CC0 1.0 Public domain dedication).

MaxQuant Analysis Spreadsheet output from the label-free quantification analysis using the MaxQuant software. The spreadsheet contains the details of the identification and quantitation of all proteins across the 21 biological samples. Click here for additional data file. Copyright: © 2018 Leung F et al. 2018 Data associated with the article are available under the terms of the Creative Commons Zero "No rights reserved" data waiver (CC0 1.0 Public domain dedication).

## Discussion

In this study, a comprehensive proteomic analysis of 21 tissues from CC and EC tumours, as well as benign endometriotic precursors was performed. The identification of almost 9000 unique proteins represents the most in-depth proteomic profiles of these gynaecological specimens to date. In fact, the workflow used has generated one of the largest datasets to date with the Q-Exactive Plus. As a comparison, in a study delineating the proteomes of pancreatic tissue specimens from controls and Type 1 diabetic patients, approximately 5500 unique proteins were identified using a similar workflow on the Q-Exactive Plus ( Liu *et al.*, 2016).

Although isotope-based labeling methods are the gold standard for quantitative proteomics, LFQ is becoming increasingly utilized due to its simplicity and practicality. Unlike labeling methods, LFQ avoids extra pre-analytical steps and can be applied to any type of sample. This is especially relevant in clinical samples that cannot be metabolically labeled using standard labeling techniques. Furthermore, recent advances with the MaxLFQ algorithm in the MaxQuant software have greatly increased the accuracy and robustness of LFQ ( Cox *et al.*, 2014). In this study, quality assessment demonstrated that proteomic data was consistent across all samples with respect to intracohort variation and housekeeping protein expression. Furthermore, correlation and clustering analysis without enrichment demonstrated that samples separated into relatively distinct clusters within their respective cohorts.

The ability of label-free quantitative proteomics to accurately recapitulate CC and EC was further highlighted by the correlation of the proteomic data with clinical IHC markers. Whereas known markers specific to the serous and mucinous subtypes were not identified almost none of the CC and EC samples, markers specific to CC and EC were identified almost exclusively in their respective subtypes. A caveat here is that our proteomic analysis will not capture specific expression patterns (such as focal or diffuse) that are commonly used in IHC-based differential diagnosis of ovarian tumours ( Kaspar & Crum, 2015). On the other hand, proteomic profiling may reveal insights that IHC-based diagnoses are unable to as the former considers protein expression across the entirety of the tumour while the latter interrogated localized areas. For example, the expression of *HNF1B* in one of the EC samples (sample EC4) may indicate a mixed CC/EC origin in that tumour due to the fact that *HNF1B* is often used to rule in a diagnosis of CC. Retrospective analysis of such ‘discordant’ tumours would be useful to determine if these tumours contained any areas of CC-like histology. Additionally, re-examination of sample EC18 via histopathology revealed more mucinous-like features which was reflected in its proteomic profile being discordant with the other EC samples. In these regards, proteomic profiling may thus enhance IHC-based diagnoses by offering a macroscopic view of ovarian tumours that would otherwise be missed.

Finally, bioinformatic analyses revealed that the CC and EC proteomes are potentially more informative than genomic data with regards to markers potentially implicated in disease pathogenesis. The CC proteome was characterized by muscle- and cytoskeleton-related processes, while the EC proteome was represented by cell junction- and extracellular matrix-related processes. Further analysis with the disease signatures revealed novel contributions from MET, α6β4 integrin and retinoic acid pathways for CC. This is the first study to identify these associations as possible avenues of pathogenesis from endometriosis, but further functional studies will need to be performed to elaborate their true roles in tumourigenesis. Nevertheless, such distinctions may be indicative of the different underlying biology and mechanisms that contribute to development of CC and EC from EMT. As a result, each subtype could be targeted for their key pathogenic mechanisms instead of the standard platinum-based chemotherapy administered to all OvCa patients. Future studies should thus expand on the use of proteomic-based profiling of endometriosis-associated cancers in order to provide novel insights into the etiology and pathogenesis of the diseases, which in turn, will affect diagnosis and treatment of these cancers.

In summary, this study has generated proteomic data for endometriosis and its associated (ovarian) cancers. Overall, we have demonstrated that not only is our dataset robust and comprehensive, but it is also reflective of the molecular profiles of the various diseases. Clustering analysis revealed unique expression patterns within the cohorts and that proteomic profiling may serve as a more accurate representation than genomic profiling. As well, disease signature analyses have demonstrated that each cancer subtype is characterized by distinct markers which can be exploited for further insight into the etiology of each subtype as well as identification of novel therapeutic targets.

## Data availability

The data referenced by this article are under copyright with the following copyright statement: Copyright: © 2018 Leung F et al.

Data associated with the article are available under the terms of the Creative Commons Zero "No rights reserved" data waiver (CC0 1.0 Public domain dedication). 

The output of the MaxQuant analyses is available in the spreadsheet ‘MaxQuant Analysis’ along with an interpretive guide (‘MaxQuant Analysis Guide’). Additional files or raw data can be provided upon reasonable request.

**Dataset 1: MaxQuant Analysis Guide –** An interpretive guide for the spreadsheet output from the MaxQuant analysis. 10.5256/f1000research.13863.d193746 ( Leung *et al.*, 2018a)

**Dataset 2: MaxQuant Analysis –** Spreadsheet output from the label-free quantification analysis using the MaxQuant software. The spreadsheet contains the details of the identification and quantitation of all proteins across the 21 biological samples. 10.5256/f1000research.13863.d193821 ( Leung *et al.*, 2018b)

## Supplementary material

**Supplementary Table 1 –** Differential proteins identified in CC and EC compared to EMT and END.

**Supplementary Table 2 –** Overrepresented ontologies identified with enrichment analysis of Gene Ontology (GO) annotations.

**Supplementary Table 3 –** Differential proteins identified with significant increased expression from END to EMT to EC.

**Supplementary Table 4 –** Differential proteins identified with significant increased expression from END to EMT to CC.

**Supplementary Figure 1 –** Summary of number of proteins identified overall and within the patient cohorts. The error bars represent standard deviation of protein identified with at least two unique peptides within each patient cohort.

**Supplementary Figure 2 –** Identified proteins categorized according to the number of peptide hits associated with each protein identification. An identified protein was defined as any protein with a non-zero normalized LFQ value in at least one patient sample.

**Supplementary Figure 3 –** Conservation of levels of ribosomal biogenesis and assembly proteins across the 21 biological replicates. The protein intensity levels are derived from the label-free quantification intensities generated from MaxQuant.

**Supplementary Figure 4 –** Clustering analysis of concordant proteins across the cancer and control cohorts.

**Supplementary Figure 5 –** Expression levels of ARID1A-related proteins (A) and PIK3CA/PTEN-related proteins (B) across the cancer and control cohorts.

**Supplementary Figure 6 –** ‘Disease signatures’ for CC (A) and EC (B) derived from identifying proteins with progressively increased expression from END to EMT to EC/CC.
