# Supplementary material for: Unraveling endometriosis-associated ovarian carcinomas using integrative proteomics
Source: F1000Res. 2018 Jun 20;7:189. Originally published 2018 Feb 14. [Version 2] doi: 10.12688/f1000research.13863.2 (PMC5915760; doi:10.12688/f1000research.13863.2)
Supplement: Supplementary file 3 [file f1000research-7-16667-s0002.tgz › a0dd836c-909c-4a90-b1f2-9bc61089f0a8.pdf]

**Supplementary Table 1 – Differential proteins identified in CC and EC compared to EMT and END.**

| Gene Name | q-value[a] |
|---|---|
| **Clear Cell Carcinoma** | |
| SPD2B | 0.00374 |
| AGRN | 0.00323 |
| ISLR | 0.00123 |
| HSPB6 | 0.00162 |
| OPLA | 0.00689 |
| LAMA5 | 0.00988 |
| CLIC2 | 0.00338 |
| TGFI1 | 0.00106 |
| HS74L | 0.00150 |
| SDPR | 0.00876 |
| ADH1B | 0.00119 |
| APOE | 0.00761 |
| GNAI2 | 0.00633 |
| LDHB | 0.00936 |
| TPM2 | 0.00142 |
| ANXA6 | 0.00587 |
| ODPA | 0.00397 |
| HCK | 0.00564 |

| | |
|---|---|
| VIME | 0.00236 |
| LEG1 | 0.00598 |
| PGFRB | 0.00115 |
| LYAG | 0.00367 |
| PPGB | 0.00610 |
| CH60 | 0.00248 |
| ODB2 | 0.00175 |
| ACTN1 | 0.00146 |
| NID1 | 0.00600 |
| KPYM | 0.00557 |
| GNS | 0.00813 |
| DESP | 0.00831 |
| BGAL | 0.00995 |
| DESM | 0.00131 |
| VINC | 0.00329 |
| RCC1 | 0.00983 |
| AOC1 | 0.00149 |
| CSRP1 | 0.00175 |
| FLNA | 0.00096 |
| MYL9 | 0.00742 |
| IVD | 0.00608 |

| | |
|---|---|
| S10A4 | 0.00264 |
| LMOD1 | 0.00123 |
| CGL | 0.00609 |
| TSP2 | 0.00078 |
| ADDA | 0.00240 |
| MYH11 | 0.00087 |
| GLRX1 | 0.00667 |
| MAP1B | 0.00102 |
| LIMS1 | 0.00263 |
| PDLI4 | 0.00088 |
| SUOX | 0.00601 |
| CNN1 | 0.00200 |
| CAD11 | 0.00907 |
| TPM4 | 0.00462 |
| ACTC | 0.00149 |
| TAGL | 0.00165 |
| CAV1 | 0.00602 |
| ACY1 | 0.00862 |
| CALD1 | 0.00087 |
| ZO1 | 0.00324 |
| AIMP1 | 0.00549 |

| | |
|---|---|
| TRAP1 | 0.00113 |
| ACACA | 0.00117 |
| SNTB2 | 0.00796 |
| RAB31 | 0.00403 |
| FHL1 | 0.00124 |
| FHL3 | 0.00328 |
| KGP1 | 0.00924 |
| FLNC | 0.00121 |
| RFTN1 | 0.00457 |
| PGM5 | 0.00310 |
| MYLK | 0.00445 |
| ZYX | 0.00120 |
| DPYL2 | 0.00718 |
| PTGIS | 0.00241 |
| AOC3 | 0.00169 |
| ANO6 | 0.00248 |
| GRAP1 | 0.00859 |
| PDLI3 | 0.00079 |
| TNS2 | 0.00594 |
| KANK2 | 0.00209 |
| SCMC1 | 0.00828 |

| | |
|---|---|
| PTRF | 0.00172 |
| OLFL1 | 0.00127 |
| MRRP1 | 0.00398 |
| KDEL2 | 0.00834 |
| PHLB1 | 0.00987 |
| SETD7 | 0.00532 |
| PALLD | 0.00088 |
| GGH | 0.00825 |
| LPP | 0.00127 |
| ISOC1 | 0.00847 |
| T3HPD | 0.00862 |
| PLIN4 | 0.00883 |
| MYADM | 0.00764 |
| SRBS1 | 0.00195 |
| RAB34 | 0.00405 |
| E41L1 | 0.00595 |
| ES8L2 | 0.00559 |
| SIAE | 0.00202 |
| MCCB | 0.00417 |
| PDLI7 | 0.00244 |
| PARVA | 0.00591 |

| | |
|---|---|
| CA123 | 0.00407 |
| EHD2 | 0.00228 |
| LMCD1 | 0.00234 |
| RAI14 | 0.00810 |
| TLN1 | 0.00398 |
| SERC | 0.00246 |
| FKBP7 | 0.00890 |
| CLIC4 | 0.00185 |
| IF2B2 | 0.00815 |
| ADSV | 0.00984 |
| TXNRD1 | 0.00917 |
| TPSB2 | 0.00852 |
| ASAP1 | 0.00932 |
| SPTAN1 | 0.00527 |
| EIF4G2 | 0.00252 |
| EZR | 0.00605 |
| HCLS1 | 0.00938 |
| TNS1 | 0.00367 |
| ESYT2 | 0.00599 |
| ANK2 | 0.00835 |
| ANTXR2 | 0.00870 |

| | |
|---|---|
| CLUH | 0.00412 |
| LONP1 | 0.00116 |
| TPM1 | 0.00124 |
| Q8WU40 | 0.00945 |
| **Endometrioid Carcinoma** | |
| HSPB6 | 0.00039 |
| MYPT1 | 0.00557 |
| CLIC2 | 0.00403 |
| TGFI1 | 0.00280 |
| WDR1 | 0.00467 |
| AL1A2 | 0.00627 |
| SVIL | 0.00410 |
| SDPR | 0.00031 |
| ADH1B | 0.00231 |
| FINC | 0.00807 |
| MYL1 | 0.00553 |
| PROS | 0.01000 |
| PGS2 | 0.00254 |
| TPM2 | 0.00258 |
| ANXA6 | 0.00910 |
| ITA5 | 0.00538 |

| | |
|---|---|
| RRAS | 0.00268 |
| CH60 | 0.00268 |
| ACTN1 | 0.00374 |
| ETFA | 0.00743 |
| DESM | 0.00269 |
| VINC | 0.00591 |
| MIME | 0.00588 |
| CSRP1 | 0.00188 |
| FLNA | 0.00218 |
| TGM2 | 0.00269 |
| TENX | 0.00400 |
| AT2B4 | 0.00368 |
| SYTC | 0.00268 |
| LMOD1 | 0.00208 |
| GNA11 | 0.00810 |
| ECHM | 0.00296 |
| HSP74 | 0.00880 |
| TSP2 | 0.00258 |
| FBN1 | 0.00197 |
| MYH11 | 0.00246 |
| GGT5 | 0.00235 |

| | |
|---|---|
| PBX1 | 0.00956 |
| MDHM | 0.00374 |
| SYIC | 0.00315 |
| MUC18 | 0.00436 |
| ENTP1 | 0.00792 |
| LRBA | 0.00998 |
| SSDH | 0.00997 |
| CNN1 | 0.00284 |
| AFAD | 0.00339 |
| CAD11 | 0.00840 |
| CAD13 | 0.00386 |
| CH10 | 0.00844 |
| ACTA | 0.00806 |
| ACTC | 0.00269 |
| TCPB | 0.00909 |
| FBLN2 | 0.00261 |
| DHSO | 0.00939 |
| TAGL | 0.00342 |
| AKA12 | 0.00291 |
| CAV1 | 0.00249 |
| CALD1 | 0.00260 |

| | |
|---|---|
| AMPE | 0.00804 |
| AHNK | 0.00505 |
| FBLN3 | 0.00419 |
| TRAP1 | 0.00236 |
| CSTF3 | 0.00784 |
| SNTB2 | 0.00476 |
| RAB31 | 0.00800 |
| FHL1 | 0.00244 |
| FLNC | 0.00463 |
| SPRL1 | 0.00554 |
| RFTN1 | 0.00345 |
| NAA25 | 0.00547 |
| ZYX | 0.00342 |
| PTGIS | 0.00371 |
| H2B2E | 0.00848 |
| AOC3 | 0.00653 |
| PDLI3 | 0.00076 |
| TSSC1 | 0.00649 |
| LR16A | 0.00918 |
| TNS2 | 0.00291 |
| KANK2 | 0.00036 |

| | |
|---|---|
| PTRF | 0.00200 |
| OLFL1 | 0.00342 |
| PACS1 | 0.00462 |
| SYDE1 | 0.00260 |
| HEAT3 | 0.00985 |
| PODN | 0.00536 |
| PHLB1 | 0.00545 |
| AEBP1 | 0.00740 |
| PALLD | 0.00252 |
| ASM3B | 0.00850 |
| LPP | 0.00345 |
| FERM2 | 0.00289 |
| H2A1J | 0.00645 |
| NIPS1 | 0.00245 |
| AASD1 | 0.00549 |
| SRBS1 | 0.00805 |
| CT027 | 0.00861 |
| SIAE | 0.00309 |
| PDLI7 | 0.00470 |
| ACS2L | 0.00338 |
| EHD2 | 0.00266 |

| | |
|---|---|
| SYLC | 0.00658 |
| MRC2 | 0.00469 |
| FBLN5 | 0.00241 |
| SYNP2 | 0.00270 |
| TLN1 | 0.00272 |
| SERC | 0.00206 |
| GPC6 | 0.00921 |
| FKBP7 | 0.00644 |
| CLIC4 | 0.00750 |
| COG4 | 0.00853 |
| LAMA4 | 0.00751 |
| EMILIN1 | 0.00751 |
| C1R | 0.00327 |
| TNS1 | 0.00231 |
| EFEMP2 | 0.00828 |
| LTBP2 | 0.00227 |
| RAB8B | 0.00883 |
| PPL | 0.00820 |
| TPM1 | 0.00265 |

[a]Bonferroni-Hochberg corrected (FDR = 0.01)
