# Supplementary material for: Unraveling endometriosis-associated ovarian carcinomas using integrative proteomics
Source: F1000Res. 2018 Jun 20;7:189. Originally published 2018 Feb 14. [Version 2] doi: 10.12688/f1000research.13863.2 (PMC5915760; doi:10.12688/f1000research.13863.2)
Supplement: Supplementary file 4 [file f1000research-7-16667-s0003.tgz › c10c4be1-b0ed-4d8f-a0aa-e525f2801e16.pdf]

Supplementary Table 2 – Overrepresented ontologies identified with enrichment analysis of Gene Ontology (GO) annotations.

Annotation	GO class[a]	Fold enrichment	q-value[b]
**Clear Cell Carcinoma**			
Homotypic cell-cell adhesion	BP	91.51	4.64E-07
Actin filament-based process	BP	15.41	2.54E-05
Actin cytoskeleton organization	BP	16.17	1.58E-04
Muscle contraction	BP	30.94	5.67E-08
Muscle system process	BP	25.59	3.02E-07
Cytoskeletal protein binding	MF	11.61	2.18E-07
Cell-substrate adherens junction	CC	23.3	4.96E-10
Cell-substrate junction	CC	23.01	5.68E-10
Actin cytoskeleton organization	CC	19.43	3.50E-09
Adherens junction	CC	14.74	6.51E-09
**Endometrioid Carcinoma**			
Collagen binding	MF	27.46	3.38E-03
Actin filament binding	MF	26.43	2.05E-06
Extracellular matrix constituent	MF	23	7.99E-03
Cytoskeletal protein binding	MF	6.84	8.07E-05
Adherens junction	CC	8.62	3.39E-08
Anchoring junction	CC	8.4	4.97E-08
Actin cytoskeleton organization	BP	12.08	1.08E-05
Extracellular matrix organization	BP	9.46	1.70E-03
Extracellular structure organization	BP	9.43	1.74E-03
Biological adhesion	BP	5.24	5.51E-04

[a]BP = biological process; MF = molecular function; CC = cellular component
[b]Bonferroni-Hochberg corrected (FDR = 0.01)
