# Supplementary material for: Unraveling endometriosis-associated ovarian carcinomas using integrative proteomics
Source: F1000Res. 2018 Jun 20;7:189. Originally published 2018 Feb 14. [Version 2] doi: 10.12688/f1000research.13863.2 (PMC5915760; doi:10.12688/f1000research.13863.2)
Supplement: Supplementary file 5 [file f1000research-7-16667-s0004.tgz › 8cd2e6cb-593d-4de5-8c70-2c47ecc2e728.pdf]

Supplementary Table 3 – Differential proteins identified with significant increased expression from END to EMT to EC.							
**Protein ID**	**ProbNull**			**P value**		**Fold Change**	
	**All comparisons**	**EC vs. EMT**	**EMT vs. END**	**EC vs. EMT**	**EMT vs. END**	**EC vs. EMT**	**EMT vs. END**
**Increase from END to EMT to EC**							
ENPP4		0.2993	0.4327	0.0344	0.0124	2.87	2.77
**Increase from END to EMT**							
GLSK	0.1319			0.5957	0.0011	0.76	3.72
Q5H9A7	0.3044			0.6839	0.0064	0.73	3.65
A0A087WVF8	0.3427			0.6269	0.0040	1.27	2.68
H7C285	0.3708			0.7302	0.0131	0.81	2.87
PLOD2	0.3757			0.3406	0.0069	0.45	2.85
MYO1F	0.3792			0.4324	0.0109	0.66	2.51
LGMN	0.3922			0.6084	0.0184	0.76	1.82
E7ENQ1	0.3922			0.2102	0.0022	0.50	7.19
GALNS	0.4067			0.9270	0.0134	0.96	2.30
NDK8	0.4371			0.7538	0.0254	0.77	3.13
NGAL	0.4381			0.9166	0.0204	0.91	1.69
VATE1	0.4423			0.4465	0.0239	0.78	1.57
GILT	0.4524			0.4172	0.0244	0.58	5.19
OXLA	0.4698			0.2475	0.0114	0.34	4.36

STXB2	0.4921			0.6147	0.0251	1.29	3.31
**Increase from EMT to EC**							
PLSI	0.3353			0.0000	0.1194	85.60	0.73
ELMO3	0.4807			0.0000	0.0589	155.27	0.43
SERC	0.1047			0.0000	0.7918	25.24	3.00
ARHGG	0.3461			0.0004	0.2319	14.94	0.68
CAH8	0.1863			0.0005	0.5885	26.53	2.08
LAD1	0.3526			0.0006	0.2028	16.23	0.63
DPCD	0.3580			0.0007	0.1650	6.62	1.87
HIP1R	0.1893			0.0007	0.6073	9.11	1.20
IRF6	0.2132			0.0007	0.5167	5.20	1.90
TRAP1	0.2200			0.0012	0.5309	3.94	1.46
A0A075B6H3	0.1207			0.0013	0.3152	4.07	3.13
PHAR4	0.4314			0.0014	0.0921	5.47	1.27
CF132	0.3681			0.0014	0.1977	9.89	1.42
DSG2	0.2211			0.0015	0.5505	8.91	1.78
L2GL2	0.3627			0.0015	0.2764	15.31	2.27
PUS7	0.4296			0.0017	0.0957	7.79	1.33
PRSS8	0.3586			0.0017	0.2942	35.84	1.55
B4DKV7	0.3401			0.0018	0.3323	76.43	1.74
RHG08	0.2795			0.0019	0.4264	7.81	0.89

EFGM	0.1758			0.0019	0.8700	3.55	1.50
MYO5B	0.3831			0.0023	0.2269	15.69	0.49
HTR5B	0.2043			0.0025	0.7535	6.15	2.73
RGPA1	0.3910			0.0025	0.1631	8.94	1.27
TYW5	0.2138			0.0026	0.7069	6.57	2.12
F5H1S9	0.4900			0.0026	0.0737	3.81	0.98
CLN5	0.2907			0.0027	0.4327	5.87	0.96
MYH14	0.4957			0.0027	0.0723	8.20	0.96
CD2AP	0.2090			0.0028	0.7571	4.21	1.27
NIPS1	0.1872			0.0028	0.9182	2.92	1.63
AT2C1	0.3593			0.0028	0.3274	6.62	0.67
MCM2	0.1904			0.0031	0.9362	12.79	1.12
RNZ2	0.4068			0.0031	0.1434	3.87	1.33
KPB1	0.2178			0.0032	0.7447	6.39	2.46
IPO4	0.3765			0.0032	0.3055	5.33	1.69
MSX1	0.1646			0.0034	0.8011	4.71	1.38
DHSO	0.4508			0.0035	0.0972	3.43	0.85
SIAE	0.4110			0.0035	0.1439	9.83	0.67
A0A0C4DFM0	0.4902			0.0037	0.0778	5.91	0.59
SYIC	0.3376			0.0039	0.3829	2.73	1.06
FUMH	0.3974			0.0039	0.2721	2.68	0.99

ZN687	0.2960			0.0041	0.4587	5.38	1.15
UMPS	0.4067			0.0043	0.2204	3.12	1.52
ACY1	0.3463			0.0044	0.3784	12.45	2.48
TM102	0.1790			0.0044	0.7059	5.64	1.24
NS1BP	0.3464			0.0045	0.3790	15.16	2.03
KLH14	0.1804			0.0046	0.4631	4.28	4.09
RT14	0.2538			0.0046	0.6386	5.89	1.49
CIB1	0.4091			0.0048	0.2546	10.15	0.78
F134C	0.3904			0.0049	0.3119	8.01	0.80
K7EKI8	0.3617			0.0049	0.3612	4.54	1.54
RT10	0.1841			0.0049	0.5800	4.50	2.01
K7ES20	0.2230			0.0049	0.8622	4.05	2.24
PRCC	0.4406			0.0052	0.1209	4.77	1.55
SYTC	0.1873			0.0052	0.7241	2.86	1.55
WDR11	0.3599			0.0052	0.3685	3.43	0.82
VIP1	0.4144			0.0053	0.2304	5.38	0.92
COG6	0.2503			0.0055	0.7115	3.52	1.77
A0A087WTV6	0.4429			0.0055	0.1208	5.82	1.47
ASM3B	0.2575			0.0057	0.6768	15.00	4.37
F120B	0.2505			0.0064	0.7586	4.46	1.52
LAMC2	0.1980			0.0064	0.6232	5.08	2.62

ESRP1	0.1983			0.0065	0.8636	12.79	3.51
AAGAB	0.2290			0.0066	0.9455	4.36	2.28
PVRL1	0.3412			0.0069	0.4174	7.81	0.47
A0A0A0MS45	0.4131			0.0071	0.2920	3.79	1.11
A0A0A0MRM8	0.4141			0.0071	0.2895	5.26	1.57
SMC2	0.2304			0.0071	0.9833	4.44	1.39
X6RHX1	0.3801			0.0073	0.3575	4.91	1.66
ACD10	0.4286			0.0077	0.2000	5.34	0.70
SH23A	0.2088			0.0079	0.4671	5.30	2.81
A2ML1	0.2099			0.0081	0.9634	5.91	2.07
HOOK2	0.4324			0.0081	0.1765	4.37	0.99
PKP3	0.4284			0.0081	0.2542	8.23	1.17
ZO2	0.2405			0.0084	0.9514	3.97	1.70
CASP8	0.2136			0.0087	0.6921	7.16	4.55
FAD1	0.2876			0.0088	0.6183	4.81	2.18
NEUL	0.2939			0.0089	0.5845	3.58	1.36
WSDU1	0.4346			0.0090	0.2014	4.91	0.56
XRN2	0.4835			0.0091	0.0979	3.04	0.95
KLC4	0.4189			0.0092	0.3034	2.62	1.19
D3DSE6	0.4367			0.0094	0.1898	3.37	1.26
TACD2	0.4364			0.0094	0.2209	15.59	0.53

NUFP2	0.4186			0.0097	0.3091	3.61	0.69
CGL	0.4162			0.0097	0.3141	9.24	1.22
MT21A	0.4271			0.0098	0.2888	3.04	0.97
PAX8	0.2939			0.0098	0.6128	5.74	2.21
F5H4M0	0.4913			0.0098	0.0943	4.69	0.68
FUK	0.4925			0.0098	0.0935	4.60	2.39
RAB25	0.3903			0.0100	0.3623	5.99	2.27
UPAR	0.2220			0.0101	0.8549	5.98	1.61
SYLC	0.3870			0.0101	0.3686	2.57	1.11
DHTK1	0.3874			0.0102	0.3684	9.09	1.62
MED19	0.4465			0.0102	0.1558	3.41	0.61
ZMYM2	0.4460			0.0102	0.1579	4.41	0.74
B4DJV2	0.4399			0.0102	0.2015	2.55	0.80
COG7	0.4405			0.0102	0.1894	6.59	3.26
GLYC	0.2982			0.0103	0.6042	4.70	2.97
AP1M2	0.2777			0.0104	0.7392	11.20	1.10
ATHL1	0.2246			0.0105	0.9785	5.72	3.42
MARC1	0.2912			0.0106	0.6572	5.42	2.49
D6RF44	0.4700			0.0108	0.1176	8.98	0.36
LRBA	0.4324			0.0109	0.2876	7.80	1.31
MFSD5	0.2273			0.0109	0.9359	3.43	1.87

SYAP1	0.3364			0.0111	0.4718	3.94	1.02
NAA25	0.2603			0.0112	0.9074	4.06	1.51
F5H0B0	0.3308			0.0113	0.4891	3.69	1.29
UBE3C	0.4391			0.0115	0.2743	3.07	1.58
MOC2B	0.4451			0.0116	0.2369	3.15	0.92
1433S	0.2709			0.0120	0.8566	11.33	0.91
E7EMW7	0.4756			0.0123	0.1182	4.50	1.45
EVPL	0.3893			0.0123	0.3818	6.66	0.54
ATPF1	0.2354			0.0123	0.6316	3.33	1.21
COG3	0.2621			0.0124	0.9905	3.55	1.46
PAPOA	0.3524			0.0125	0.4514	2.81	0.81
SAP18	0.4873			0.0126	0.1064	3.53	0.86
PSB3	0.4523			0.0126	0.1752	2.39	1.16
UBR3	0.4388			0.0129	0.2940	21.52	3.64
IFT25	0.4561			0.0129	0.1633	5.51	0.46
CAPON	0.3186			0.0130	0.5681	3.18	1.29
AATC	0.4715			0.0130	0.1284	3.02	1.03
DFFB	0.4522			0.0131	0.2220	5.80	0.68
MDHM	0.4423			0.0132	0.2880	2.61	1.11
DHX57	0.4666			0.0133	0.1393	7.82	1.03
PLAK	0.4649			0.0134	0.1441	11.17	0.93

GNA1	0.4456			0.0135	0.2821	4.84	0.89
NALP2	0.2444			0.0138	0.5279	5.46	2.00
PROM1	0.2445			0.0138	0.9337	26.21	0.85
CDC23	0.3395			0.0141	0.5068	7.34	2.75
STX6	0.4440			0.0142	0.2956	5.04	1.24
GALE	0.4226			0.0143	0.3412	3.47	1.06
TTI1	0.4315			0.0145	0.3258	7.42	0.80
KIFA3	0.3035			0.0145	0.7179	2.49	1.95
RM20	0.4569			0.0145	0.2478	7.33	1.55
DHAK	0.4589			0.0148	0.2348	2.48	1.14
RUVB1	0.4548			0.0148	0.2685	2.42	1.17
STRBP	0.3270			0.0148	0.5761	4.76	1.13
DI3L1	0.3070			0.0149	0.7087	8.21	3.35
TPD52	0.3446			0.0151	0.5062	8.76	2.47
E7EQR4	0.2517			0.0151	0.2595	3.32	1.84
AKAP9	0.4596			0.0151	0.2446	6.61	1.13
ANM5	0.4453			0.0152	0.3028	2.37	1.18
OGT1	0.2941			0.0153	0.8183	2.78	0.83
M0QYA2	0.4930			0.0154	0.1122	3.09	0.54
MCM5	0.3176			0.0154	0.6535	7.24	0.75
TSR1	0.4689			0.0154	0.1549	2.89	0.96

MCE1	0.4790			0.0159	0.1356	2.99	0.88
CH10	0.2915			0.0159	0.8631	2.44	1.07
TRI32	0.4699			0.0160	0.1585	5.38	0.59
F5H1Z6	0.4883			0.0160	0.1214	9.73	0.55
A0A0A0MTG1	0.4652			0.0163	0.2363	6.19	0.83
MET13	0.4730			0.0164	0.1541	4.21	1.09
G3V5V3	0.4582			0.0164	0.2810	8.94	2.53
ANC2	0.2597			0.0165	0.7393	2.55	1.29
FOLH1	0.3450			0.0165	0.5315	7.46	0.78
XPO5	0.4675			0.0166	0.1887	2.82	0.90
GCC2	0.3019			0.0167	0.8122	5.23	2.66
PAF1	0.4714			0.0169	0.1681	3.78	1.37
AG10B	0.2899			0.0172	0.9570	2.98	2.17
E7ER68	0.4654			0.0172	0.2644	4.44	0.70
CAMP3	0.3061			0.0173	0.8059	4.92	1.90
MET14	0.4696			0.0174	0.2374	15.12	5.44
E7ETZ4	0.2651			0.0174	0.7972	2.79	1.76
RT29	0.4716			0.0175	0.1833	7.84	0.28
A0A087WVI0	0.2659			0.0176	0.3844	3.12	1.99
HDHD3	0.4743			0.0178	0.1707	4.01	0.74
ACAD8	0.4567			0.0180	0.3037	3.74	0.50

NSUN6	0.2682			0.0180	0.8301	3.02	2.40
ARP3B	0.2967			0.0182	0.9361	4.95	1.51
PRKRA	0.4276			0.0186	0.3652	2.66	1.12
RT11	0.3067			0.0186	0.8569	3.70	1.18
FABD	0.3498			0.0187	0.5576	3.41	0.82
E7ENA2	0.4986			0.0187	0.1216	16.05	1.72
DHX16	0.4835			0.0187	0.1507	2.54	0.68
ETFA	0.3246			0.0189	0.7325	2.07	1.09
AL2CL	0.2743			0.0191	0.7733	2.48	1.97
ADRM1	0.4415			0.0192	0.3448	2.28	1.08
SUMO2	0.2747			0.0192	0.8437	5.59	1.24
S14L2	0.4310			0.0196	0.3670	3.67	0.60
TELO2	0.3832			0.0197	0.4608	2.97	0.78
ARFG3	0.4630			0.0199	0.3067	3.44	0.66
ANS1A	0.4295			0.0202	0.3742	4.05	3.54
THTR	0.4523			0.0206	0.3354	3.43	0.83
A8K7Q2	0.3433			0.0209	0.6692	7.03	0.60
FA84B	0.4849			0.0212	0.1954	4.97	0.67
PTPRK	0.5000			0.0215	0.1365	2.75	0.84
AVL9	0.3285			0.0215	0.7984	5.75	1.14
MANBA	0.2872			0.0216	0.4125	3.79	2.54

ALAT2	0.3193			0.0217	0.8809	3.67	1.59
RM23	0.4842			0.0219	0.2622	3.74	1.06
LYAG	0.2894			0.0220	0.8476	3.44	3.20
SYTM	0.3496			0.0220	0.6598	5.28	2.90
ERF3A	0.3584			0.0222	0.6028	2.55	1.39
DPH2	0.4183			0.0225	0.4118	3.45	1.61
ANKH1	0.4900			0.0227	0.2168	5.08	0.72
SUGP1	0.4911			0.0231	0.2331	4.36	0.66
RBM47	0.2945			0.0231	0.6252	9.13	1.25
MIEAP	0.2946			0.0231	0.6281	4.65	2.84
CNNM4	0.3290			0.0232	0.8586	5.92	0.73
POGK	0.2957			0.0233	0.9766	4.23	3.55
INVO	0.2960			0.0234	0.5843	6.22	4.36
KI21A	0.4931			0.0236	0.2284	5.96	0.84
IPYR	0.4617			0.0237	0.3402	2.51	0.76
WIPF2	0.4847			0.0243	0.2924	3.56	1.64
SYRC	0.3002			0.0243	0.6541	2.21	1.41
ATR	0.4037			0.0244	0.4594	6.56	0.46
RAM	0.3970			0.0244	0.4779	6.50	1.48
ZC3HF	0.3844			0.0245	0.5200	2.44	1.36
WIBG	0.3013			0.0245	0.5071	2.72	1.69

SETB1	0.3019			0.0246	0.9380	3.03	2.43
DCTP1	0.3030			0.0248	0.7361	3.78	1.41
ACS2L	0.3032			0.0249	0.7554	3.22	2.45
COG2	0.3037			0.0250	0.1590	2.35	1.83
LR16A	0.3428			0.0251	0.8132	8.02	1.21
MCCB	0.4880			0.0255	0.2948	3.29	0.81
CUL4B	0.4998			0.0256	0.2225	2.40	0.98
PTBP3	0.4119			0.0257	0.4518	6.21	1.54
SET	0.4910			0.0261	0.2921	2.17	1.06
RAI3	0.3663			0.0261	0.6670	6.71	2.02
CH60	0.4535			0.0264	0.3738	2.09	1.07
CAYP1	0.4908			0.0265	0.2965	21.12	0.88
MCP	0.3995			0.0266	0.4965	11.61	2.56
GCN1L	0.3961			0.0267	0.5095	2.16	1.10
F8I2	0.4557			0.0269	0.3728	3.57	1.29
SYMC	0.3925			0.0271	0.5294	2.40	1.15
XPO7	0.4932			0.0272	0.2964	2.24	1.27
ST14	0.3714			0.0272	0.6631	5.90	1.26
ZDH13	0.3786			0.0273	0.6119	3.90	0.88
MCM7	0.3473			0.0274	0.8595	6.82	1.42
D2HDH	0.4903			0.0277	0.3082	4.73	0.87

GLYG2	0.3910			0.0280	0.5529	3.94	1.80
CELR1	0.3504			0.0280	0.8536	3.73	1.47
SG2A1	0.3504			0.0281	0.8552	15.57	0.85
GUAD	0.3889			0.0281	0.5662	17.00	1.29
RRP12	0.4385			0.0282	0.4141	3.26	1.00
MPPD2	0.3624			0.0284	0.7670	3.93	1.61
DDAH1	0.4934			0.0285	0.3069	3.20	1.08
MET2A	0.3195			0.0286	0.3577	3.82	5.26
TPMT	0.3196			0.0286	0.9721	2.46	1.50
A16L1	0.4167			0.0287	0.4682	5.49	1.36
KDIS	0.3457			0.0288	0.9383	5.12	2.15
ERBB2	0.4985			0.0289	0.2963	4.55	2.07
SYQ	0.3616			0.0290	0.7915	2.29	1.10
SRPK2	0.4739			0.0297	0.3563	2.88	1.16
ADRO	0.4184			0.0298	0.4739	3.44	1.33
S12A2	0.3631			0.0301	0.8130	2.96	0.79
PPBN	0.3491			0.0302	0.9817	64.14	6.06
WAC	0.3885			0.0305	0.6287	9.53	2.48
ARFP2	0.4197			0.0307	0.4791	3.79	0.87
BPHL	0.4894			0.0309	0.3323	2.51	1.71
AIMP1	0.3743			0.0310	0.7512	2.36	1.35

FAS	0.3297			0.0311	0.8504	2.69	1.75
RT25	0.4889			0.0311	0.3350	3.45	1.07
F8VSA1	0.3670			0.0313	0.8204	4.72	1.65
DSC2	0.3999			0.0314	0.5731	6.82	0.60
G3V174	0.3901			0.0315	0.6436	3.23	1.23
CBPD	0.3709			0.0324	0.8217	4.65	0.71
TSTD1	0.4965			0.0328	0.3292	8.33	2.01
ERO1A	0.3363			0.0328	0.3665	2.39	1.92
MCM6	0.3365			0.0329	0.9067	4.22	1.66
FCL	0.4192			0.0333	0.5101	2.48	1.92
COMT	0.3379			0.0333	0.9434	2.85	2.52
A0A087WVU5	0.4082			0.0343	0.5821	5.37	3.10
HOOK1	0.3768			0.0344	0.8283	3.88	1.09
GSHB	0.4807			0.0344	0.3703	2.05	1.14
PUR6	0.4327			0.0344	0.4772	2.17	0.99
FXL18	0.4681			0.0347	0.3961	2.95	1.28
ECHP	0.3766			0.0349	0.8484	4.03	1.33
SPF45	0.4373			0.0354	0.4719	3.20	1.33
SYEP	0.3896			0.0360	0.7657	2.27	1.19
TXND9	0.4153			0.0364	0.5796	2.25	1.25
SPIT1	0.4153			0.0373	0.5989	6.04	0.53

IPO9	0.4053			0.0376	0.6826	2.62	2.04
FREM2	0.3914			0.0379	0.8049	3.72	1.39
PSMD7	0.4093			0.0379	0.6591	2.15	1.64
AACS	0.4365			0.0382	0.5016	8.38	0.84
KLK11	0.3561			0.0384	0.9377	25.69	0.82
LCHN	0.3576			0.0389	0.5482	3.81	1.60
MK13	0.3592			0.0394	0.3569	2.90	1.43
J3KNC0	0.3905			0.0396	0.8611	6.49	0.37
MIPEP	0.3610			0.0399	0.6336	2.22	1.36
MED27	0.4754			0.0401	0.4121	3.20	0.46
KLC2	0.4030			0.0402	0.7663	3.00	0.84
CRYM	0.3626			0.0404	0.8817	4.82	1.61
Q5JRU2	0.3896			0.0411	0.9224	3.34	1.54
AOC1	0.3910			0.0414	0.9151	10.91	0.68
BCCIP	0.4787			0.0416	0.4133	2.19	1.11
SARG	0.4186			0.0417	0.6731	4.23	0.93
C1QBP	0.4680			0.0430	0.4473	1.95	0.91
CHCH5	0.4232			0.0436	0.6815	4.42	1.64
RT07	0.4666			0.0445	0.4612	4.99	0.74
TCPG	0.4538			0.0447	0.5053	2.08	1.22
PCKGM	0.3770			0.0451	0.6306	3.69	2.20

H0YMZ1	0.4470			0.0452	0.5428	2.20	1.33
BROX	0.4435			0.0453	0.5641	2.88	1.60
GIPC1	0.3786			0.0456	0.9106	4.38	0.72
IDHC	0.3788			0.0456	0.9380	2.97	2.35
HMCS1	0.3789			0.0457	0.0960	3.91	1.27
IDE	0.4002			0.0457	0.9943	2.12	0.96
PROM2	0.4130			0.0459	0.8222	3.15	1.81
AIMP2	0.4017			0.0460	0.9694	2.18	1.24
PRS8	0.3821			0.0468	0.8882	1.98	1.78
BOLA3	0.4492			0.0469	0.5550	3.23	0.77
CB054	0.3829			0.0470	0.7377	3.18	0.89
UCHL5	0.3840			0.0474	0.7081	2.42	2.75
SPB5	0.4479			0.0483	0.5855	8.93	0.73
M0R3D4	0.3874			0.0486	0.6310	3.58	1.38
HTSF1	0.4212			0.0487	0.8176	3.12	1.84
OXSM	0.4233			0.0489	0.8029	5.33	3.45
NRCAM	0.4976			0.0491	0.4130	8.83	1.15
GGH	0.3894			0.0493	0.2462	3.41	2.77
E7ERS3	0.4783			0.0496	0.4645	2.67	1.08
M0QWZ7	0.4849			0.0498	0.4475	2.27	1.34
TRM1L	0.4642			0.0498	0.5167	4.67	1.80
