# Supplementary material for: Unraveling endometriosis-associated ovarian carcinomas using integrative proteomics
Source: F1000Res. 2018 Jun 20;7:189. Originally published 2018 Feb 14. [Version 2] doi: 10.12688/f1000research.13863.2 (PMC5915760; doi:10.12688/f1000research.13863.2)
Supplement: Supplementary file 6 [file f1000research-7-16667-s0005.tgz › 07a77a0f-8729-4d8f-89ac-fe7e5cfb3294.pdf]

Supplementary Table 4 – Differential proteins identified with significant increased expression from END to EMT to CC.							
Protein ID	ProbNull			P value		Fold Change	
	All comparisons	CC vs. EMT	EMT vs. END	CC vs. EMT	EMT vs. END	CC vs. EMT	EMT vs. END
**Increase from END to EMT to CC**							
ENPP4		0.2993	0.4327	0.0344	0.0124	2.87	2.77
**Increase from END to EMT**							
APOE	0.4349			0.1492	0.0001	0.58	6.81
PLOD2	0.1880			0.4971	0.0003	0.69	13.36
FIBG	0.4999			0.1286	0.0005	0.48	8.01
E7EVW7	0.2376			0.4593	0.0007	0.79	3.92
KV119	0.2902			0.4355	0.0016	0.64	8.85
GLRX1	0.1982			0.1507	0.0017	1.85	4.93
CATZ	0.2614			0.5923	0.0025	0.82	3.94
CFAH	0.2972			0.5602	0.0036	0.77	4.59
DAPK1	0.2630			0.9670	0.0041	1.02	5.16
PLOD1	0.2652			0.9041	0.0050	0.95	3.78
OXLA	0.3211			0.5525	0.0051	0.64	12.06
CO3	0.3843			0.4021	0.0055	0.69	4.27
HV320	0.4889			0.2381	0.0055	0.41	11.49
GLSK	0.2848			0.2703	0.0059	2.18	9.50
CTHR1	0.4796			0.2556	0.0061	0.40	13.22
LCAT	0.4561			0.2885	0.0061	0.57	5.46

E9PF32	0.3329			0.5558	0.0064	0.69	7.36
F13A	0.3233			0.6040	0.0068	0.80	3.99
PLEK	0.4168			0.3684	0.0073	0.39	26.51
HTAI2	0.3940			0.4175	0.0075	0.63	5.80
CO5	0.3241			0.6920	0.0086	0.84	3.74
GILT	0.3141			0.6859	0.0089	1.28	6.30
PROP	0.4474			0.3436	0.0094	0.55	6.48
Q5H9A7	0.3604			0.5629	0.0097	0.63	11.11
A0A075B6I8	0.4629			0.3335	0.0106	0.59	4.83
B4E1Z4	0.3968			0.4888	0.0111	0.71	4.19
PPIC	0.3463			0.9890	0.0122	1.01	5.91
C1QC	0.3476			0.8996	0.0123	1.07	4.45
F5GZZ9	0.4792			0.3305	0.0125	0.59	4.55
HPTR	0.3496			0.8405	0.0128	0.90	4.63
CAH2	0.3544			0.1072	0.0130	2.14	3.63
CSPG2	0.3854			0.1609	0.0170	1.96	3.54
C9JGI3	0.3961			0.7966	0.0189	1.16	4.69
NBEL2	0.4057			0.8824	0.0209	1.09	4.78
NGAL	0.3967			0.9645	0.0219	0.96	8.55
A0A087WZW8	0.4436			0.5682	0.0223	0.53	17.56
LGMN	0.4126			0.8557	0.0228	1.10	4.06
GSH0	0.4217			0.7223	0.0232	0.88	2.49

TFR1	0.4495			0.5564	0.0232	0.76	3.22
TPM3	0.4175			0.4961	0.0242	1.40	3.46
CH3L1	0.4129			0.9038	0.0252	0.90	8.94
KV113	0.4449			0.6215	0.0262	0.69	6.67
R39L5	0.4245			0.9876	0.0264	1.02	11.54
ATRN	0.4252			0.3218	0.0267	1.53	2.87
CO4B	0.4237			0.8613	0.0277	0.92	3.40
FSTL1	0.4199			0.9488	0.0286	0.97	3.28
HPT	0.4312			0.9263	0.0287	1.07	5.61
HABP2	0.4330			0.9062	0.0293	1.06	3.55
EM55	0.4471			0.6704	0.0295	0.78	4.09
BRE1B	0.4468			0.6904	0.0302	0.82	3.42
A0A0A0MTQ6	0.4355			0.8198	0.0305	0.82	8.03
PARP4	0.4981			0.4606	0.0307	0.77	2.38
LV302	0.4903			0.5085	0.0330	0.50	11.54
BPI	0.4777			0.5687	0.0337	0.57	9.99
VATE1	0.4486			0.5672	0.0341	1.23	2.32
NNMT	0.4475			0.8211	0.0342	0.85	5.50
COPZ1	0.4494			0.9655	0.0344	1.02	2.44
THMS2	0.4535			0.8397	0.0356	1.12	3.89
CDD	0.4486			0.8924	0.0360	0.89	7.75
HGFL	0.4853			0.5715	0.0362	0.71	4.04

LIMA1	0.4896			0.5802	0.0380	0.82	2.30
FA49B	0.4724			0.2052	0.0407	1.52	2.08
SN	0.4875			0.6984	0.0422	0.77	4.71
AACT	0.4939			0.2533	0.0460	2.22	4.51
**Increase from EMT to CC**							
AIF1L	0.4349			0.0000	0.0991	22.21	0.51
AOC1	0.0015			0.0000	0.6849	494.15	1.43
ERBB2	0.1144			0.0000	0.7056	22.19	0.84
K7EL76	0.0021			0.0000	0.7695	25.42	1.15
SERC	0.1324			0.0000	0.6321	86.77	0.72
HS74L	0.0029			0.0000	0.7152	22.27	1.19
PAX8	0.3234			0.0000	0.2665	24.39	0.57
CGL	0.1570			0.0000	0.5419	109.39	0.63
ADSV	0.4379			0.0000	0.0998	97.21	0.26
XDH	0.2286			0.0000	0.3988	182.18	0.46
ES8L2	0.1908			0.0000	0.4665	15.12	0.70
ACY1	0.2711			0.0000	0.3394	39.06	0.53
NS1BP	0.4993			0.0000	0.0546	56.59	0.22
AGRIN	0.0867			0.0000	0.8195	10.00	0.91
OPLA	0.3667			0.0000	0.2147	10.01	0.58
OGDHL	0.4397			0.0000	0.1002	61.69	0.27
TRAP1	0.2127			0.0000	0.4286	5.06	0.78

RIR2B	0.3969			0.0001	0.1755	16.09	0.47
NAPSA	0.0119			0.0001	0.2415	64.69	2.63
MRRP1	0.4113			0.0001	0.1563	5.79	0.61
MCCB	0.3234			0.0001	0.2760	5.71	0.68
HNF1B	0.1485			0.0001	0.6075	9.49	0.79
SGK2	0.0151			0.0001	0.7055	13.30	1.22
IF2B2	0.4455			0.0001	0.0938	16.46	0.37
ALAT2	0.0168			0.0001	0.7379	11.75	1.19
LYAG	0.0643			0.0001	0.9489	5.98	0.98
B4DKV7	0.2994			0.0001	0.3106	52.00	0.43
K7EIG1	0.0175			0.0001	0.3088	13.45	1.75
GRB7	0.4381			0.0001	0.1159	33.59	0.30
LAMA5	0.0894			0.0002	0.8412	12.51	0.90
AACS	0.4019			0.0002	0.1748	19.92	0.41
SYHM	0.3593			0.0002	0.2333	10.27	0.55
RCC1	0.3559			0.0002	0.2380	6.13	0.63
SPIT1	0.3747			0.0002	0.2135	12.79	0.50
CC50A	0.3689			0.0002	0.2223	12.71	0.50
ODB2	0.4462			0.0002	0.1022	3.68	0.62
ACD10	0.4758			0.0002	0.0668	12.35	0.35
FA84B	0.3228			0.0002	0.2849	26.73	0.46
NDRG1	0.2529			0.0003	0.3833	7.70	0.67

GUAD	0.2321			0.0003	0.4169	84.11	0.45
ORNT1	0.3842			0.0003	0.2052	14.04	0.47
BIG2	0.4325			0.0003	0.1387	5.81	0.54
WNK1	0.4231			0.0003	0.1533	6.43	0.54
CSTN1	0.4399			0.0003	0.1257	15.28	0.38
EFNA1	0.3248			0.0003	0.2892	10.92	0.55
ANXA4	0.2195			0.0003	0.4456	9.88	0.67
SSDH	0.4880			0.0004	0.0631	5.54	0.46
PRSS8	0.3188			0.0004	0.2998	33.86	0.42
SYAP1	0.2324			0.0004	0.4287	6.52	0.70
LFTY1	0.3728			0.0004	0.2290	112.25	0.25
VAS1	0.3091			0.0005	0.3179	9.96	0.57
E41L1	0.3109			0.0006	0.3193	3.56	0.73
E9PEJ6	0.0872			0.0006	0.9645	19.49	0.97
RTKN	0.1546			0.0006	0.6970	8.51	0.81
LR16A	0.0455			0.0007	0.7561	6.17	1.15
GCC2	0.4024			0.0007	0.1969	9.27	0.48
PFKAP	0.3125			0.0007	0.3204	4.26	0.70
MET	0.1518			0.0007	0.7154	8.01	0.83
CAYP1	0.4505			0.0007	0.1252	21.14	0.30
PAR6B	0.0481			0.0007	0.6833	6.11	1.20
K7EJE8	0.4234			0.0008	0.1709	2.88	0.69

ARHGG	0.3376			0.0008	0.2901	13.07	0.50
JAM1	0.2854			0.0008	0.3629	39.78	0.43
IMPA2	0.4601			0.0008	0.1099	47.63	0.19
PKP3	0.4658			0.0009	0.0951	16.69	0.29
CLPX	0.1628			0.0009	0.6956	6.22	0.83
ZN217	0.0538			0.0009	0.3437	8.42	1.69
FA84A	0.0973			0.0009	0.9898	10.54	0.99
COX17	0.2441			0.0009	0.4370	5.50	0.71
GCSP	0.1393			0.0009	0.7876	68.08	0.75
LDHB	0.3972			0.0009	0.2128	3.28	0.68
LRBA	0.3291			0.0010	0.3078	4.35	0.68
CH60	0.2601			0.0010	0.4111	2.94	0.79
QOR	0.4688			0.0010	0.0952	3.70	0.55
COBL1	0.0593			0.0011	0.2481	6.92	1.81
RDH10	0.0597			0.0011	0.5233	40.61	1.86
GYS1	0.0613			0.0011	0.7602	4.50	1.13
GALE	0.1906			0.0012	0.6067	8.68	0.74
TOM34	0.4765			0.0012	0.0854	5.26	0.45
NEUR1	0.4248			0.0012	0.1815	16.40	0.36
VAV2	0.1772			0.0013	0.6782	4.90	0.84
UNG	0.4752			0.0013	0.0917	19.82	0.24
B1ALH6	0.0696			0.0014	0.8367	12.82	1.15

LDHD	0.2314			0.0015	0.4925	8.45	0.67
FOLR1	0.4706			0.0015	0.1123	16.85	0.28
SYWC	0.2632			0.0016	0.4269	6.10	0.67
LAMC1	0.0728			0.0016	0.7834	4.77	1.12
TPD53	0.2223			0.0016	0.5200	11.59	0.65
CLN5	0.3289			0.0016	0.3250	5.37	0.63
E7EQR4	0.0736			0.0016	0.2820	3.88	1.50
B4GT1	0.0742			0.0016	0.4365	22.28	1.96
AL3B1	0.0745			0.0016	0.3610	5.53	1.55
CING	0.2078			0.0018	0.5887	8.68	0.72
T2FA	0.4290			0.0018	0.1882	7.72	0.46
CADH1	0.4687			0.0019	0.1273	25.97	0.24
J3KS22	0.4820			0.0019	0.0929	3.27	0.56
K0513	0.0801			0.0019	0.5709	6.20	1.34
ESRP1	0.0810			0.0019	0.8773	13.01	1.12
RT25	0.2894			0.0021	0.3961	4.10	0.71
E41L5	0.4025			0.0021	0.2313	5.27	0.56
GRAP1	0.2316			0.0021	0.5221	2.86	0.82
A0A0A0MRM8	0.3735			0.0021	0.2742	3.21	0.69
BAIP2	0.3024			0.0022	0.3782	8.70	0.58
IMPA3	0.3389			0.0022	0.3246	5.87	0.60
IVD	0.4171			0.0022	0.2127	2.65	0.70

AP1M2	0.1587			0.0022	0.8203	17.79	0.83
LYPA1	0.3763			0.0023	0.2728	3.40	0.68
RDH13	0.1423			0.0023	0.8877	9.52	0.91
IF2B3	0.0904			0.0024	0.4173	7.24	1.60
HIP1R	0.3337			0.0024	0.3369	13.58	0.48
PUR1	0.2118			0.0024	0.6225	4.40	0.81
GNS	0.0958			0.0027	0.3942	3.35	1.36
HTR5B	0.3621			0.0027	0.3017	4.92	0.61
PYGL	0.1488			0.0028	0.9004	3.11	0.96
DLP1	0.1736			0.0028	0.8036	19.01	0.81
DSG2	0.3450			0.0029	0.3297	6.11	0.59
KCRS	0.3440			0.0029	0.3311	5.19	0.62
CO4A2	0.1002			0.0029	0.9583	10.39	1.04
AATC	0.3633			0.0029	0.3038	4.26	0.64
COA3	0.1014			0.0030	0.4874	4.35	1.36
RPGF6	0.1842			0.0030	0.7806	10.39	0.82
ARHG5	0.3812			0.0031	0.2806	6.39	0.54
KAD4	0.2636			0.0032	0.4808	4.59	0.72
IPO4	0.2532			0.0032	0.5084	3.61	0.77
PTCD3	0.3327			0.0032	0.3545	3.11	0.73
ADRO	0.2665			0.0032	0.4757	4.53	0.72
PLIN2	0.2310			0.0033	0.5922	34.17	0.56

MFHA1	0.4994			0.0034	0.0899	8.61	0.31
E7ENA2	0.2981			0.0034	0.4156	3.84	0.71
G5EA36	0.3503			0.0036	0.3345	4.99	0.62
ANXA4	0.2808			0.0036	0.4551	30.10	0.46
GRP75	0.2381			0.0037	0.5871	2.86	0.84
C19L1	0.4924			0.0037	0.1185	6.57	0.39
A0A087WWM1	0.2620			0.0038	0.5071	15.46	0.57
A0A087WTV6	0.2612			0.0038	0.5110	4.96	0.72
SCAM3	0.3949			0.0039	0.2747	5.78	0.55
ELMO3	0.4627			0.0040	0.1746	94.90	0.14
PHAR4	0.4791			0.0041	0.1513	3.57	0.55
OSTP	0.2362			0.0043	0.6365	74.56	0.53
GATA	0.1235			0.0043	0.6348	4.05	1.23
FA83B	0.1243			0.0044	0.9795	7.30	1.02
MCCA	0.3716			0.0045	0.3184	3.20	0.69
CNNM4	0.1261			0.0045	0.5226	10.96	1.63
F5H0B0	0.2784			0.0046	0.4919	5.88	0.68
FARP2	0.4856			0.0047	0.1486	4.92	0.47
GGH	0.1304			0.0048	0.0770	3.95	2.26
SYNE2	0.4617			0.0049	0.1887	5.05	0.50
RBM47	0.1338			0.0050	0.5179	13.71	1.73
HOOK2	0.2507			0.0050	0.6133	5.62	0.76

GOLM1	0.1342			0.0050	0.8617	4.83	1.09
RHG29	0.1353			0.0051	0.8734	5.40	1.09
PTPRK	0.4911			0.0053	0.1490	5.32	0.45
MCP	0.1388			0.0053	0.6443	8.15	1.37
CYS1	0.1786			0.0054	0.9748	5.89	0.98
A0A0B4J2A4	0.1825			0.0054	0.9375	3.12	0.97
ACSM3	0.2803			0.0055	0.5155	6.17	0.68
KS6A1	0.3153			0.0056	0.4323	6.37	0.62
SCRIB	0.4989			0.0056	0.1385	2.99	0.58
PUR6	0.4211			0.0056	0.2612	2.71	0.69
FAKD2	0.4414			0.0056	0.2304	4.44	0.55
SLPI	0.2545			0.0057	0.6393	10.72	0.69
D6RHI9	0.1451			0.0058	0.3921	5.21	1.60
SPIT2	0.3016			0.0059	0.4704	7.45	0.62
A0A087WXX9	0.3626			0.0059	0.3548	4.76	0.62
HACL1	0.1474			0.0060	0.3521	3.53	1.48
CNKR1	0.4853			0.0060	0.1670	4.04	0.52
COA6	0.4383			0.0061	0.2414	5.57	0.51
B4DJV2	0.4634			0.0061	0.2034	2.58	0.66
OAT	0.1503			0.0062	0.9934	4.16	1.00
TFPI2	0.1511			0.0062	0.9682	11.74	1.03
BIG1	0.2563			0.0063	0.6644	3.61	0.83

NIPS1	0.1526			0.0064	0.7121	2.67	1.13
FBX50	0.2080			0.0066	0.8744	49.97	0.81
KI13B	0.3888			0.0066	0.3247	4.26	0.62
EMAL2	0.1558			0.0066	0.6848	3.66	1.19
PNPH	0.1595			0.0069	0.0873	4.12	2.35
FHR1	0.3993			0.0069	0.3133	6.44	0.53
AIMP1	0.2127			0.0073	0.8932	2.26	0.96
NFU1	0.3272			0.0073	0.4460	3.67	0.71
MDHM	0.4903			0.0073	0.1760	2.24	0.68
TFB2M	0.2236			0.0074	0.8479	3.19	0.93
A6NGP5	0.1658			0.0074	0.6351	8.32	1.41
FUMH	0.4498			0.0075	0.2426	2.37	0.70
ADAP1	0.1703			0.0078	0.1272	5.50	2.53
BCAT2	0.4815			0.0079	0.1962	2.52	0.66
E9PJD7	0.2967			0.0079	0.5512	5.77	0.70
PDK1	0.2749			0.0082	0.6762	5.70	0.78
RMD1	0.2433			0.0082	0.8100	2.76	0.92
NUP50	0.3644			0.0084	0.3926	4.00	0.66
RENR	0.1799			0.0086	0.2757	5.11	1.88
RABP1	0.2841			0.0086	0.6504	41.61	0.55
CIB1	0.3964			0.0087	0.3416	7.75	0.50
FCL	0.2763			0.0088	0.6970	3.28	0.85

NFS1	0.3324			0.0089	0.4696	2.94	0.76
ZN687	0.3322			0.0089	0.4713	6.24	0.63
RISC	0.1855			0.0091	0.1278	6.72	2.89
ASM3B	0.4590			0.0093	0.2494	11.32	0.36
THTR	0.4458			0.0095	0.2737	3.04	0.64
IPYR2	0.4671			0.0096	0.2398	2.26	0.71
ST14	0.3834			0.0098	0.3790	7.04	0.54
BGAL	0.1929			0.0098	0.1180	2.90	1.85
GNA1	0.4614			0.0098	0.2517	4.13	0.55
PSB5	0.3850			0.0102	0.3819	2.70	0.73
B7Z493	0.2769			0.0102	0.7539	4.19	0.85
ASSY	0.2937			0.0102	0.6776	2.75	0.86
FUCO2	0.1994			0.0104	0.0765	4.66	2.81
LACB2	0.4177			0.0107	0.3324	4.08	0.61
ATPF1	0.2026			0.0107	0.7192	5.45	1.25
M0R208	0.3149			0.0110	0.5978	6.54	0.70
RAB9A	0.3802			0.0112	0.4053	2.65	0.74
M0QWZ7	0.3255			0.0114	0.5636	3.00	0.79
ACSF2	0.2110			0.0116	0.6046	6.06	1.41
VTCN1	0.2789			0.0120	0.8074	11.65	0.80
CHP3	0.4941			0.0124	0.2247	3.62	0.55
TBCD8	0.2957			0.0124	0.7549	7.34	0.79

NHLC3	0.2540			0.0125	0.9926	5.09	0.99
MIPEP	0.2634			0.0126	0.9002	2.80	0.95
GRPE1	0.2559			0.0126	0.9651	2.98	0.98
C9J5D1	0.4800			0.0127	0.2510	2.46	0.67
MSLN	0.2225			0.0129	0.2330	20.76	3.98
RM37	0.4590			0.0129	0.2888	3.35	0.61
DHE3	0.2252			0.0132	0.8448	3.02	1.08
NPT2B	0.3127			0.0135	0.7152	9.56	0.73
CASP7	0.4796			0.0135	0.2602	5.00	0.50
PVR	0.2630			0.0136	0.9914	4.62	0.99
IL18	0.3288			0.0137	0.6356	4.58	0.76
ETHE1	0.2772			0.0138	0.8781	3.83	0.93
YBOX3	0.2307			0.0139	0.1882	3.24	1.83
FAS	0.2333			0.0142	0.9178	3.36	1.05
GLYC	0.4510			0.0145	0.3169	6.17	0.49
RT31	0.4711			0.0146	0.2839	3.94	0.57
MAVS	0.3439			0.0147	0.5897	2.56	0.82
ISOC2	0.4327			0.0147	0.3511	2.58	0.71
LDHA	0.2376			0.0148	0.6826	2.79	1.17
PPID	0.4889			0.0148	0.2555	2.30	0.69
DLDH	0.3673			0.0152	0.5052	2.89	0.76
RBSK	0.2409			0.0152	0.3248	2.78	1.48

H9KV28	0.4446			0.0154	0.3366	2.42	0.72
EF2K	0.3961			0.0154	0.4320	12.01	0.47
RHPN2	0.2451			0.0158	0.9644	6.39	1.03
LYAM1	0.2823			0.0158	0.9421	3.58	0.96
LAMB1	0.2462			0.0159	0.7146	3.21	1.18
DCTP1	0.2874			0.0160	0.9134	4.27	0.94
MA2B2	0.2475			0.0161	0.9749	2.70	1.01
NDKB	0.3577			0.0162	0.5697	2.28	0.83
P5CR3	0.4163			0.0162	0.3984	3.49	0.66
CC134	0.4875			0.0164	0.2723	4.12	0.54
TXD12	0.3566			0.0172	0.6076	2.58	0.83
E9PPJ5	0.4887			0.0183	0.2856	3.50	0.58
PAPOA	0.4264			0.0183	0.4001	3.26	0.67
GDF15	0.2630			0.0183	0.3518	5.12	1.85
EST2	0.4459			0.0185	0.3641	15.61	0.37
GDE	0.3552			0.0186	0.6669	2.40	0.86
PLTP	0.2656			0.0187	0.2843	4.75	1.97
PDCL3	0.2680			0.0191	0.7787	2.27	1.10
CNDP2	0.3995			0.0197	0.4858	2.64	0.76
CPSM	0.2722			0.0197	0.9997	28.80	1.00
SIR5	0.4848			0.0198	0.3043	2.71	0.66
KV304	0.2729			0.0198	0.4781	3.79	1.47

CBPD	0.3189			0.0200	0.8782	6.13	0.89
RT05	0.4376			0.0200	0.3954	3.63	0.64
PDIP2	0.2745			0.0201	0.8428	2.15	1.06
MET7B	0.3384			0.0201	0.7902	3.15	0.88
WIBG	0.2751			0.0202	0.6789	3.28	1.22
4ET	0.3436			0.0202	0.7700	3.13	0.87
ISG15	0.4403			0.0206	0.3953	5.63	0.55
F8WC54	0.2783			0.0207	0.5802	3.72	1.35
F8W7C6	0.4964			0.0210	0.2937	7.88	0.41
CPT2	0.2808			0.0211	0.9096	2.81	1.05
ACD11	0.2855			0.0219	0.4055	5.01	1.75
PREP	0.2869			0.0221	0.8635	2.00	1.05
G3P	0.3782			0.0227	0.6642	1.94	0.89
XPO2	0.4556			0.0228	0.3855	2.28	0.74
MCM2	0.4747			0.0230	0.3487	8.90	0.42
PROM1	0.2935			0.0232	0.8881	37.98	1.24
NAA25	0.3810			0.0232	0.6624	2.19	0.87
CHMP3	0.2947			0.0234	0.5533	2.89	1.30
C9J167	0.3599			0.0235	0.7781	5.43	0.82
SG2A1	0.4378			0.0236	0.4337	6.24	0.55
A0A087WVM4	0.2966			0.0237	0.9185	6.54	1.08
GCSH	0.4444			0.0240	0.4218	6.98	0.52

H0Y8R1	0.2987			0.0241	0.3741	3.18	1.55
B3GN7	0.3878			0.0242	0.6512	3.68	0.78
DECR	0.3006			0.0244	0.6597	2.58	1.19
FREM2	0.3711			0.0245	0.7499	13.73	0.70
TB22A	0.3765			0.0245	0.7241	4.65	0.80
TTC4	0.3020			0.0247	0.9026	2.64	1.05
FRYL	0.3511			0.0248	0.8492	3.63	0.90
COG7	0.4112			0.0248	0.5368	2.63	0.78
MGLL	0.3047			0.0251	0.8841	3.24	1.08
LAMA3	0.4187			0.0256	0.5209	4.86	0.65
PCCB	0.4729			0.0257	0.3755	2.43	0.71
PMM2	0.4160			0.0258	0.5360	3.37	0.73
LAT1	0.3690			0.0260	0.7966	4.26	0.85
LPIN2	0.3098			0.0260	0.4378	4.15	1.61
MET2A	0.3103			0.0261	0.3239	2.83	1.56
LAMB3	0.4069			0.0266	0.6040	6.98	0.65
SUCA	0.3444			0.0266	0.9803	2.49	0.99
SYTM	0.3590			0.0268	0.8634	4.19	0.90
ESRP2	0.3892			0.0270	0.7243	4.25	0.80
ERO1A	0.3155			0.0271	0.2918	2.53	1.53
SPAG1	0.3175			0.0274	0.3304	2.68	1.52
BOLA3	0.3199			0.0279	0.3118	3.63	1.77

TTC38	0.3675			0.0286	0.8669	2.37	0.94
HDDC2	0.4432			0.0289	0.4872	2.20	0.79
ODBA	0.4337			0.0289	0.5227	2.33	0.79
MUTA	0.4261			0.0290	0.5589	2.00	0.84
1433S	0.3648			0.0290	0.8960	6.59	0.90
TPD52	0.3280			0.0294	0.5169	5.06	1.59
MAGA4	0.4289			0.0305	0.5811	3.43	0.74
AP1G1	0.3848			0.0307	0.8317	2.16	0.93
SYG	0.4321			0.0308	0.5711	2.13	0.83
PI4KB	0.4838			0.0311	0.4014	3.35	0.64
GPC5C	0.3386			0.0314	0.1428	2.85	2.03
MANBA	0.3402			0.0317	0.1343	2.92	2.10
LPPRC	0.4701			0.0317	0.4426	2.11	0.77
NID2	0.3417			0.0319	0.5927	5.38	1.49
CDCP1	0.3447			0.0325	0.1815	3.18	2.04
PHS	0.4886			0.0326	0.4047	2.18	0.75
CD2AP	0.3831			0.0328	0.8995	2.32	0.95
MTMRA	0.3467			0.0329	0.6762	2.50	1.19
ASPG	0.3474			0.0330	0.1086	4.75	3.22
CYTC	0.3474			0.0330	0.7871	2.05	1.09
EFGM	0.3774			0.0332	0.9980	2.89	1.00
MMS19	0.4454			0.0333	0.5601	1.97	0.84

ARFG3	0.4861			0.0337	0.4217	3.28	0.65
RAB2A	0.4408			0.0337	0.5958	1.98	0.85
Q5R363	0.4696			0.0344	0.4776	2.97	0.70
PRDX3	0.4586			0.0348	0.5272	1.92	0.83
FA73A	0.4898			0.0351	0.4272	4.41	0.58
PLSI	0.4482			0.0354	0.5967	8.00	0.60
H0YLX4	0.4261			0.0357	0.7443	3.96	0.81
CASP6	0.3620			0.0359	0.9057	2.25	1.04
COG3	0.4696			0.0360	0.5017	2.68	0.74
CPIN1	0.3952			0.0360	0.9270	2.25	0.97
LY75	0.3628			0.0361	0.8569	3.83	1.12
H3BRL3	0.4837			0.0362	0.4568	3.01	0.69
RM18	0.4832			0.0362	0.4586	2.21	0.76
MER34	0.3666			0.0369	0.5450	5.97	1.65
DGKK	0.3677			0.0371	0.5994	7.17	1.61
CREG1	0.4317			0.0372	0.7498	3.63	0.83
SYQ	0.4232			0.0383	0.8176	2.02	0.93
PREY	0.4444			0.0385	0.7092	2.73	0.84
STBD1	0.4237			0.0397	0.8463	2.31	0.93
VATA	0.3803			0.0397	0.1909	1.94	1.52
IDHP	0.3811			0.0399	0.5144	2.52	1.33
F5H6Z0	0.4548			0.0399	0.6825	3.31	0.79

ENOA	0.4279			0.0400	0.8325	1.82	0.94
ETFA	0.4657			0.0402	0.6129	1.84	0.86
KCMF1	0.4600			0.0406	0.6672	3.91	0.76
A0A087WUN7	0.4154			0.0407	0.9396	2.75	0.96
GGT1	0.3860			0.0409	0.5154	8.22	1.91
E7ET49	0.3891			0.0416	0.6625	3.35	1.28
FOXO1	0.4620			0.0417	0.6820	2.62	0.83
BICC1	0.3906			0.0419	0.1090	2.96	2.36
TAOK3	0.4201			0.0424	0.9833	3.08	0.99
A0A0A6YYL1	0.4819			0.0424	0.5651	3.56	0.71
VNN1	0.3939			0.0427	0.7027	10.13	1.52
HS105	0.4286			0.0427	0.8974	1.93	0.96
ACSL1	0.3943			0.0427	0.5078	3.37	1.47
ECHP	0.4742			0.0428	0.6269	5.19	0.68
TIP	0.4273			0.0429	0.9154	4.34	0.93
AP1S1	0.3993			0.0439	0.3791	2.54	1.49
LG3BP	0.4008			0.0442	0.8289	2.49	1.10
E7EMZ9	0.4923			0.0444	0.5481	5.98	0.60
R4RL2	0.4017			0.0444	0.9715	7.16	1.03
D2HDH	0.4827			0.0453	0.6337	3.95	0.73
PSME4	0.4062			0.0454	0.8639	3.70	1.11
VATG1	0.4065			0.0455	0.2877	3.06	1.79

CAH8	0.4983			0.0458	0.5453	6.52	0.58
GLT11	0.4118			0.0467	0.4482	4.51	1.75
SYEP	0.4844			0.0476	0.6867	1.90	0.88
PTER	0.4955			0.0480	0.6158	2.64	0.79
RM09	0.4185			0.0483	0.4794	3.41	1.53
PPIF	0.4195			0.0486	0.9578	3.05	1.03
TIM8A	0.4876			0.0486	0.6895	3.72	0.77
A6XND1	0.4214			0.0490	0.3124	3.83	1.97
NHRF3	0.4887			0.0493	0.6988	5.18	0.73
RM15	0.4537			0.0493	0.9129	1.96	0.96
ECH1	0.4549			0.0496	0.9116	3.02	0.94
