# Supplementary material for: Unraveling endometriosis-associated ovarian carcinomas using integrative proteomics
Source: F1000Res. 2018 Jun 20;7:189. Originally published 2018 Feb 14. [Version 2] doi: 10.12688/f1000research.13863.2 (PMC5915760; doi:10.12688/f1000research.13863.2)
Supplement: Supplementary file 7 [file f1000research-7-16667-s0006.tgz › 5c382d22-167b-4ff4-b5c7-fdbe4b709b50.pdf]

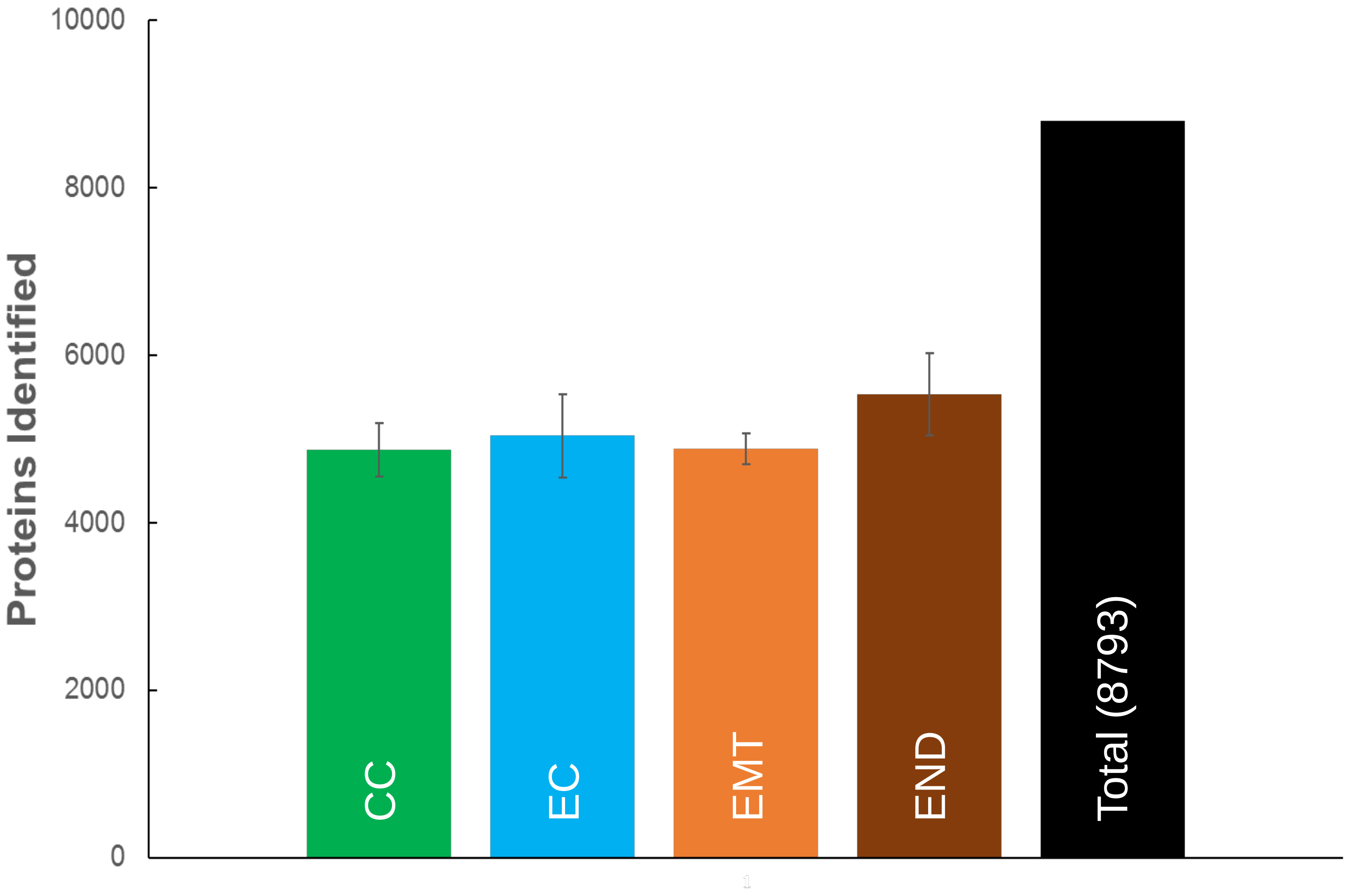

Supplementary Figure 1 – Summary of number of proteins identified overall and within the patient cohorts. The error bars represent standard deviation of protein identified with at least two unique peptides within each patient cohort.
