# Supplementary material for: Unraveling endometriosis-associated ovarian carcinomas using integrative proteomics
Source: F1000Res. 2018 Jun 20;7:189. Originally published 2018 Feb 14. [Version 2] doi: 10.12688/f1000research.13863.2 (PMC5915760; doi:10.12688/f1000research.13863.2)
Supplement: Supplementary file 8 [file f1000research-7-16667-s0007.tgz › 01f2fad2-aa3a-4f28-926f-95fe0d7cc00d.pdf]

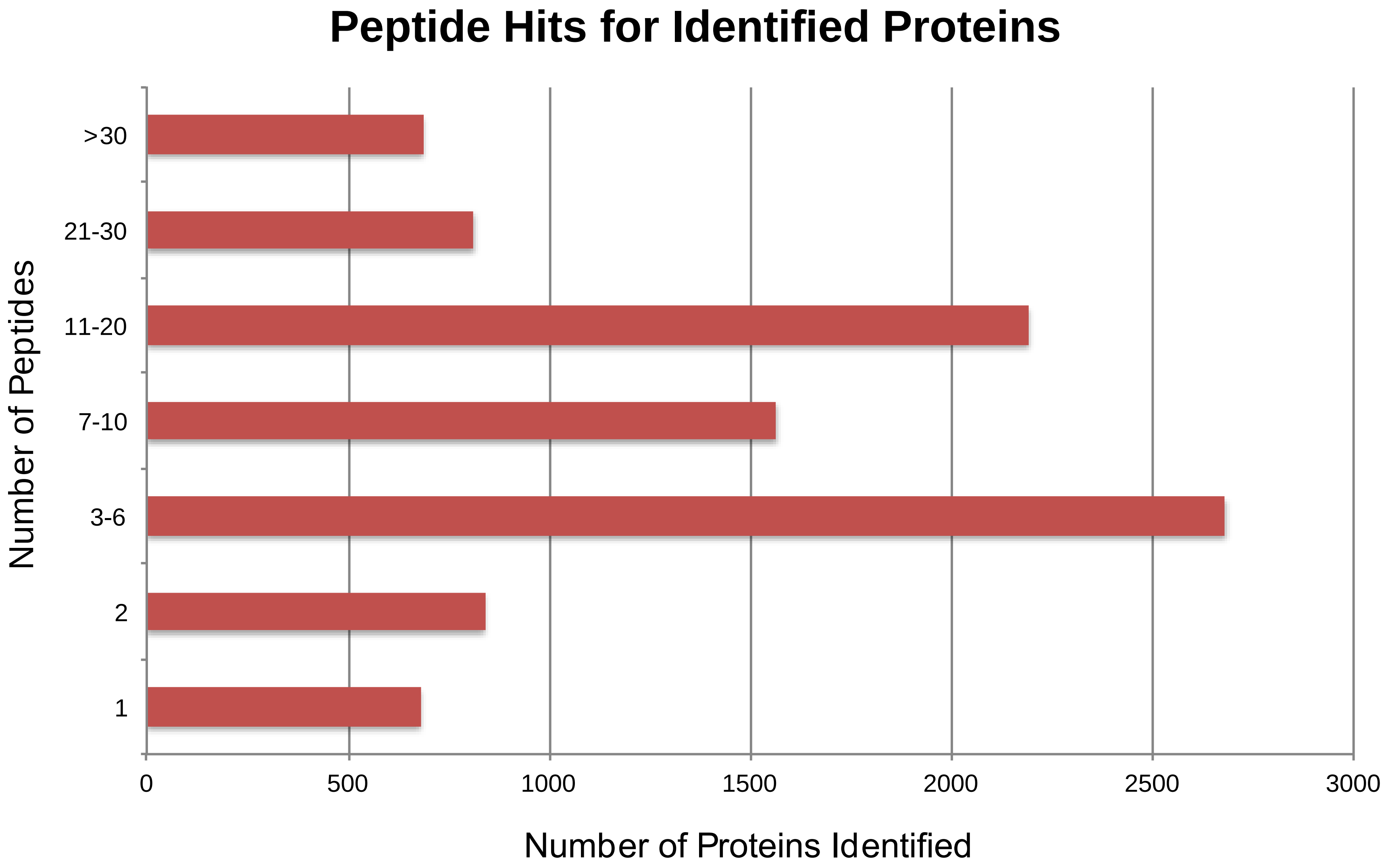

Supplementary Figure 2 – Identified proteins categorized according to the number of peptide hits associated with each protein identification. An identified protein was defined as any protein with a non-zero normalized LFQ value in at least one patient sample.
