# Supplementary material for: Unraveling endometriosis-associated ovarian carcinomas using integrative proteomics
Source: F1000Res. 2018 Jun 20;7:189. Originally published 2018 Feb 14. [Version 2] doi: 10.12688/f1000research.13863.2 (PMC5915760; doi:10.12688/f1000research.13863.2)
Supplement: Supplementary file 9 [file f1000research-7-16667-s0008.tgz › ce99261b-46b4-4158-9797-5d5a487e7ccf.pdf]

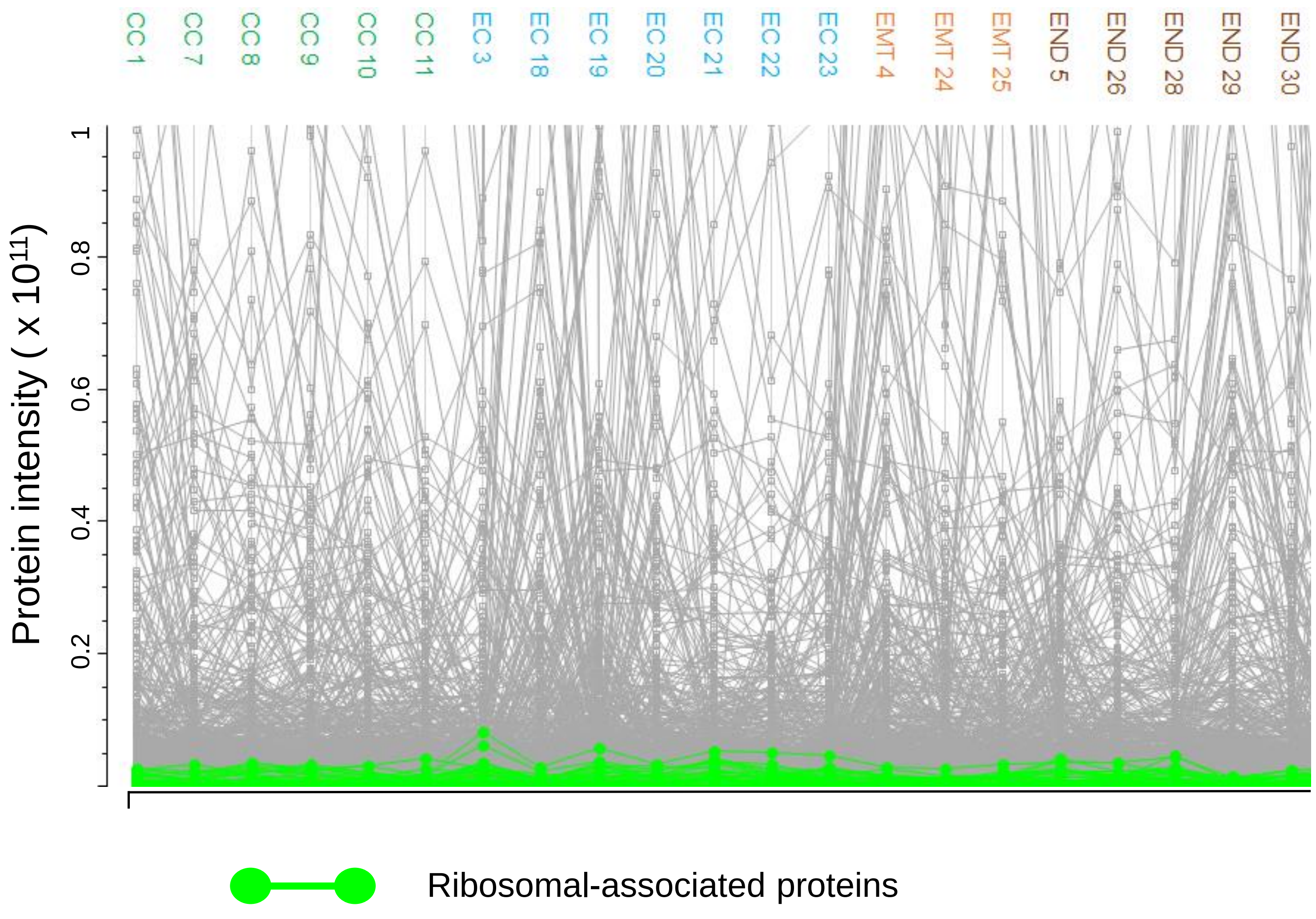

Supplementary Figure 3 – Conservation of levels of ribosomal biogenesis and assembly proteins across the 21 biological replicates. The protein intensity levels are derived from the label-free quantification intensities generated from MaxQuant.
