# Supplementary material for: Unraveling endometriosis-associated ovarian carcinomas using integrative proteomics
Source: F1000Res. 2018 Jun 20;7:189. Originally published 2018 Feb 14. [Version 2] doi: 10.12688/f1000research.13863.2 (PMC5915760; doi:10.12688/f1000research.13863.2)
Supplement: Supplementary file 10 [file f1000research-7-16667-s0009.tgz › 02d46d44-542d-4e03-b934-3a53f6e7fe64.pdf]

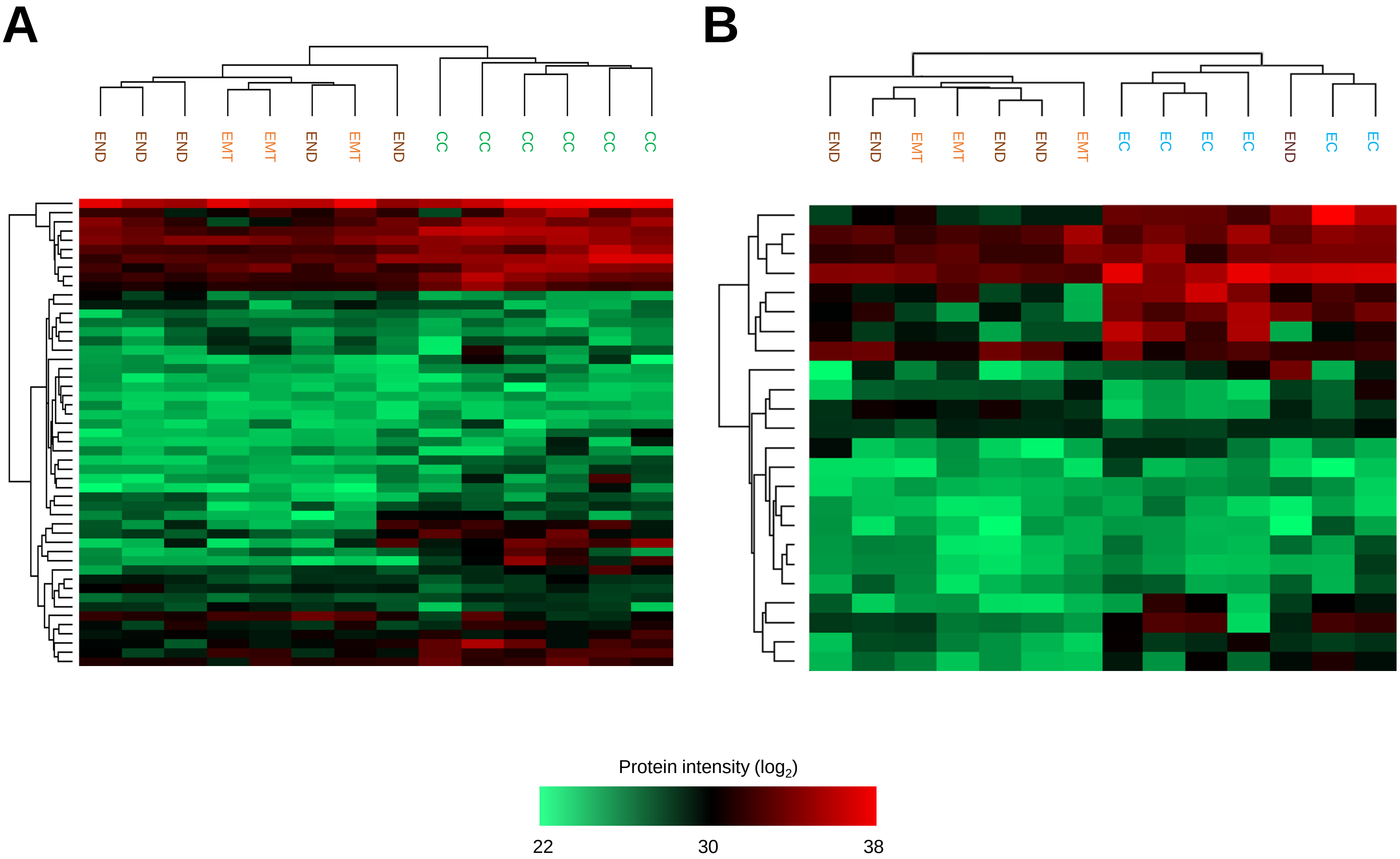


**Supplementary Figure 4 –** Clustering analysis of concordant proteins across the cancer and control cohorts.
