# Supplementary material for: Unraveling endometriosis-associated ovarian carcinomas using integrative proteomics
Source: F1000Res. 2018 Jun 20;7:189. Originally published 2018 Feb 14. [Version 2] doi: 10.12688/f1000research.13863.2 (PMC5915760; doi:10.12688/f1000research.13863.2)
Supplement: Supplementary file 11 [file f1000research-7-16667-s0010.tgz › 0d66a9d6-4f27-415c-88db-dfb9cdd82f4d.pdf]

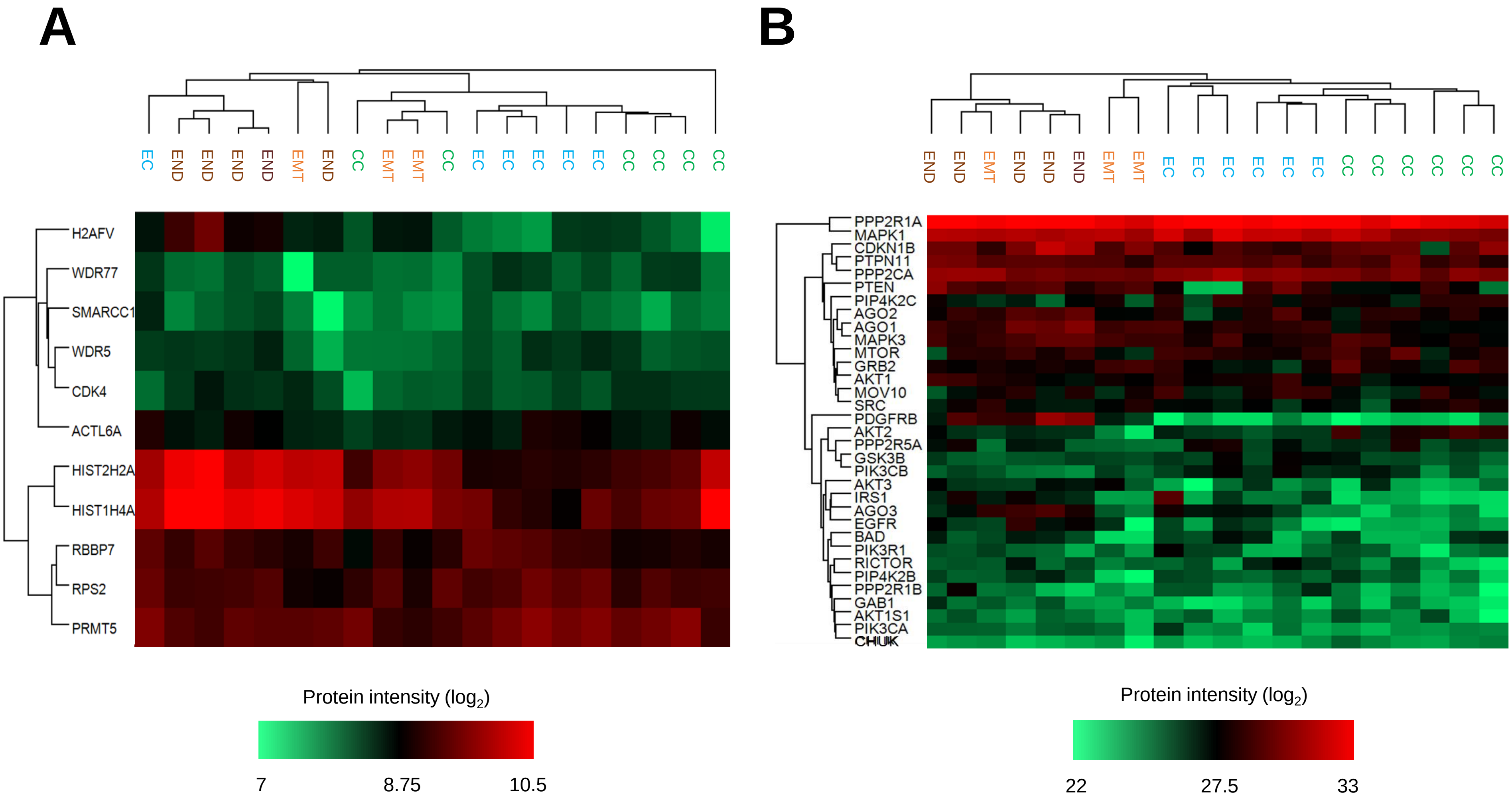

Supplementary Figure 5 – Expression levels of ARID1A-related proteins (A) and PIK3CA/PTEN-related proteins (B) across the cancer and control cohorts.
