# Supplementary material for: Unraveling endometriosis-associated ovarian carcinomas using integrative proteomics
Source: F1000Res. 2018 Jun 20;7:189. Originally published 2018 Feb 14. [Version 2] doi: 10.12688/f1000research.13863.2 (PMC5915760; doi:10.12688/f1000research.13863.2)
Supplement: Supplementary file 12 [file f1000research-7-16667-s0011.tgz › a6d312f6-11ff-440d-8709-d949e29d7102.pdf]

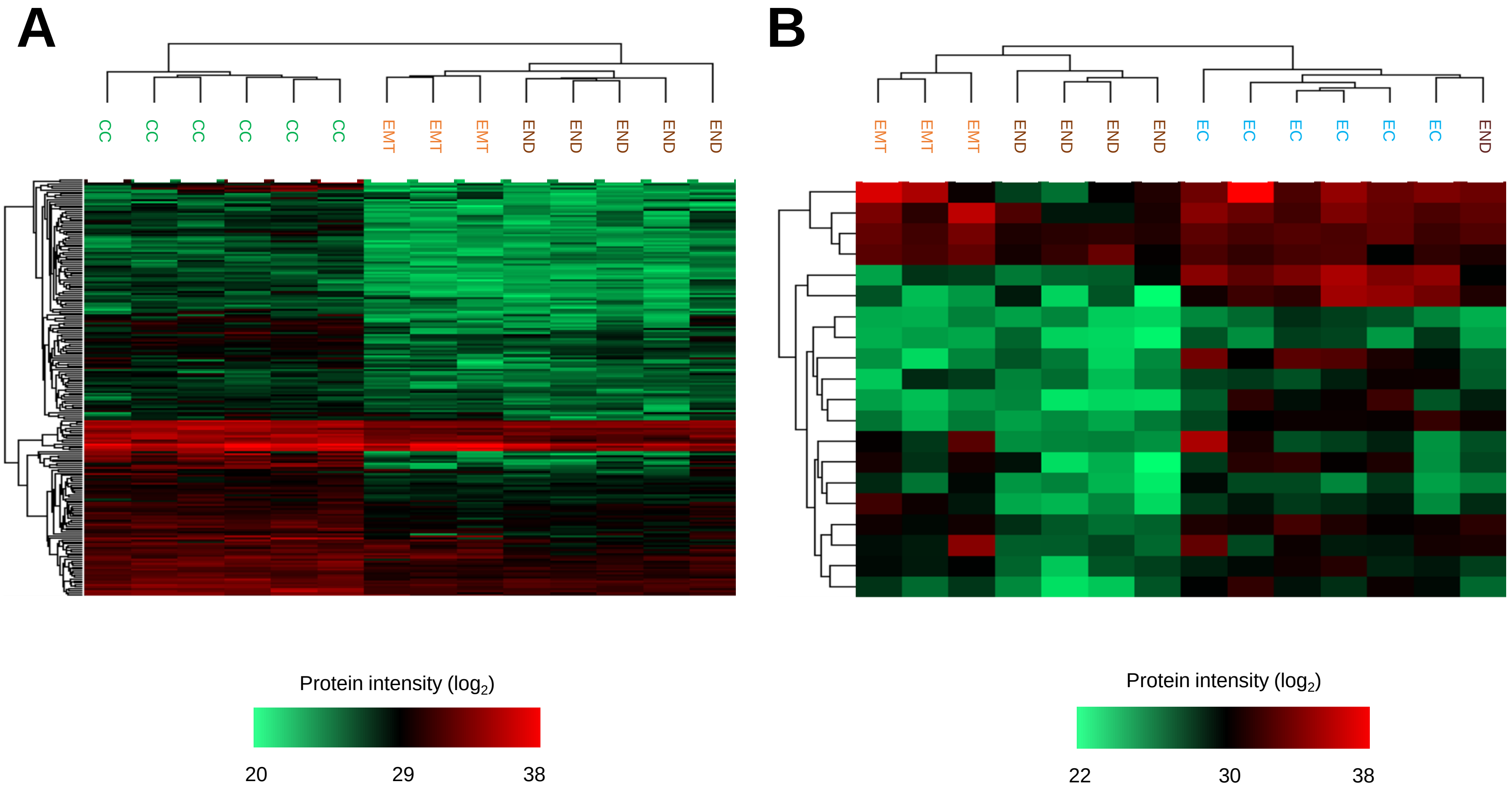

Supplementary Figure 6 – ‘Disease signatures’ for CC (A) and EC (B) derived from identifying proteins with progressively increased expression from END to EMT to EC/CC.
