# Supplementary material for: Unraveling endometriosis-associated ovarian carcinomas using integrative proteomics
Source: F1000Res. 2018 Jun 20;7:189. Originally published 2018 Feb 14. [Version 2] doi: 10.12688/f1000research.13863.2 (PMC5915760; doi:10.12688/f1000research.13863.2)
Supplement: Peer review history

# Peer review: Referee response for version 1

**DOI:** 10.5256/f1000research.15069.r32818

**Published:** 2018-04-23

**Authors:** Rajeevan Selvaratnam

**Affiliations:** 1 BayCare Health System, Tampa, FL, USA

Leung et al., has applied a proteomic profiling approach to solubilized gynecological tissue samples to delineate protein markers differentiating subtypes of non-serous ovarian cancer. Notably, these sub-types include protein markers that may differentiate endometrioid and clear cell carcinoma. Using 21 patient samples, of which 6 patients were histopathologically diagnosed with clear cell carcinoma and another 6 samples with endometrioid carcinoma, the authors have identified over 5000 proteins in the small cohorts using mass spectrometry based label-free quantitative proteomics approach. In this regard, I found Supplementary Figure 1 with the Venn diagram to be more informative than Figure 1b. It may be worthwhile to consider swapping Figure 1b with supplementary Figure 1. In general, this manuscript has many figures that nicely complement each other, including the covariance analysis presented in Figure 1c and the principal component analysis in Figure 3a.

 The experimental procedures applied here are sound. However, I am curious to know if the 1 cm ^3^ of the tissue is a randomly selected segment or purposely segmented region of the tissue that define the tumor stage. This may be important for the samples in the early tumor stages and in general where there is heterogeneity as may be the case for sample 18. Nonetheless, it appears the approach is to be a macroscopic view as the authors note and the results are encouraging. I found the strength of the study was in the ability to show the contrast between previous genomic data and the author’s proteomic findings (notably for the expression of ARI1DA, PTEN, and PIK3CA). However, the authors also indicate some closeness of their findings to previous genomic data on markers associated with clear cell carcinoma.

 Although the size of samples or cohorts may be small, these initial findings are easily appreciated and much needed to pursue further directions toward understanding non-serous ovarian cancer subtypes. Given the novelty of potential pathways implicated in clear cell carcinoma from the findings and need for further evaluation on a clinical and scientific basis, I believe this manuscript needs only minor revisions and should be accepted for indexing.

 Minor comments:

- Report centrifugation speeds in x *g* unit, as the RPM is not indicative of the force and varies with radius of the centrifuge.
- Figure 1b caption. Authors note the error bars are the standard deviation. Briefly elaborate on the standard deviation or spread of which data (e.g. within each data set based on 2 minimum peptides, or software determined, etc).

I have read this submission. I believe that I have an appropriate level of expertise to confirm that it is of an acceptable scientific standard.

# Response

**Published:** 2018-06-09

**Authors:** Vathany Kulasingam

**Affiliations:** University Health Network, Canada

Thank you for raising these important points. The caption for Figure 1b has been modified to explain that the standard deviations were calculated within each data set based on protein identifications with a minimum of 2 unique peptides. As well, Figure 1b has been switched with Supplementary Figure 1.

 Dr. Selvaratnam’s point of heterogeneity within the tissue sample is valid. It was decided that macroscopic dissection of the frozen tissue block combined with histopathological review for tumour purity of that section (>70% tumour tissue with minimal necrotic/benign tissue) was sufficient for the purposes of this study. Some degree of heterogeneity is unavoidable but as the reviewer has noted, our proteomic data does show concordance with some of the more prominent genomic markers for both endometrioid and clear cell carcinomas.

 The centrifugation units have been modified as per the reviewer’s comments.

# Peer review: Referee response for version 1

**DOI:** 10.5256/f1000research.15069.r31989

**Published:** 2018-04-03

**Authors:** Nathalie Lepage

**Affiliations:** 1 Children’s Hospital of Eastern Ontario Research Institute, Ottawa, ON, Canada

The authors of the manuscript evaluated protein content and quantitation of tissues collected from 21 patients with a range of ovarian diseases (endometriosis, endometrioid and clear cell ovarian carcinomas). They used a well-designed proteomics approach. Their findings were unique and did not exactly correlated with current knowledge from genomic analysis. Clearly additional work will be required to confirm and expand on their current findings.

 Minor comments:

1. There are some proteins that were identified only using 1 peptide (>500 proteins). The authors should elaborate of the expected protein size for these proteins and confirm that the identification based on 1 peptide is sufficient.

I have read this submission. I believe that I have an appropriate level of expertise to confirm that it is of an acceptable scientific standard.

# Response

**Published:** 2018-06-09

**Authors:** Vathany Kulasingam

**Affiliations:** University Health Network, Canada

Thank you for raising this point. We would like to clarify that although the dataset contains proteins identified only using 1 peptide, these proteins were not selected for further analyses (only those with a minimum of 2 unique peptides were investigated further). We have modified our manuscript to reflect this point. We are confident that through setting the false discovery rate (FDR) at 1% for both the peptide and protein levels, we were able to minimize false identifications even for proteins of higher molecular weight. This has been shown to be fairly resistant to false identifications even with 1 peptide identification (Gupta N & Pevzner PA. J Proteome Res 8:4173-4181 2009). The expected sizes of the proteins (identified using 1 peptide) range from approximately 10-670 kDa.
